# Design and validation of a multilevel voltage source inverter based on modular H-bridge cells

**DOI:** 10.1016/j.ohx.2023.e00452

**Published:** 2023-07-07

**Authors:** Julio Pacher, Jorge Rodas, Alfredo Renault, Magno Ayala, Leonardo Comparatore, Raul Gregor

**Affiliations:** Laboratory of Power and Control Systems, Facultad de Ingeniería, Universidad Nacional de Asunción, Paraguay

**Keywords:** Model predictive control, Modular H-bridge, Multilevel converter, SiC-Mosfet, Voltage source inverter, DC/AC converter, Digital, Power electronics, Printed circuit board design, Three-phase inverter

## Abstract

Generation, power conversion and subsequent integration of renewable energy generation systems, such as solar photovoltaic or wind, require an efficient power conversion system that can provide sufficient quality energy according to technical standards (e.g. IEEE 519–2022). In this context, this paper focuses on the analysis, design and experimental validation of a multilevel voltage source inverter (VSI) scheme based on H-bridge cells with a modular and scalable structure for its application in power electronic converter circuits. The designed and assembled experimental setup is a versatile platform for testing experimentally varied control strategies and power converter configurations, such as the number of levels (3, 5, 7 levels) and phases (single-phase or three-phase). Therefore, the hardware design process proposed for the H-bridge cell and the measurement and conditioning circuits for voltage and current signals necessary for implementing the control algorithms are explained in detail. Moreover, a quantitative analysis of the operation of the design was carried out from measurements made with the experimental platform to verify its correct operation. Among the analysed parameters, the generated harmonics level stands out, quantified by calculating the total harmonic distortion and the mean square error between the reference signals and the measured values.

## Graphical abstract

**Figure:**
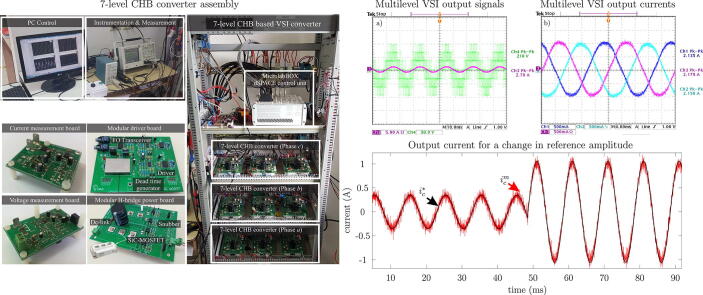

**Specifications table:**

**Table:** 

**Hardware name**	*Multilevel voltage source inverter based on modular H-bridge cells*
**Subject area**	•*Engineering and material science*
	•*Power electronics*
	•*Power converters*
**Hardware type**	•*Field measurements and sensors*
	•*Power converter*
	•*Electrical engineering*
**Closest commercial analog**	*The closest commercial equivalent would be compact three-phase multilevel power inverters. The proposed design offers an open and modular architecture for custom designs, allowing the analysis of various inverter configurations and control algorithms.*
**Open source license**	GNU General Public License (GNU GPL v3)
**Cost of hardware**	*Trigger control circuit for H-bridge: 100.82 US*$*.*
	*Power circuit for the H-bridge: 1126.93 US*$*.*
	*Voltage sensor: 30.82 US*$*.*
	*Current sensor: 37.83 US*$*.*
	*Total cost of H-bridge cell and sensors: 1246.4 US*$*.*
**Source file repository**	*Source files repository (**OSF**) write the DOI URL here. http://doi.org/10.17605/OSF.IO/ZG3RP*

## 1 Hardware in context

Multilevel converters are essential in integrating renewable energies, especially in applications such as wind farms and photovoltaic solar generation [1]. These devices enable the efficient conversion and control of the energy generated by renewable sources, facilitating their integration into the electrical grid [2]. However, multilevel converters applied in renewable energies present challenges due to these energy sources’ variability and intermittent nature. Adapting to fluctuations in power generation, maintaining efficiency, and ensuring the quality of the delivered energy to the electrical grid are critical aspects [3]. Furthermore, multilevel converters introduce higher complexity compared to conventional two-level converters. They require a more intricate hardware structure and sophisticated control algorithms to ensure proper performance and efficient switching between voltage levels. Despite these challenges, multilevel converters offer significant advantages. They enable higher energy efficiency, resulting in lower operating costs. They also have a longer component lifespan, leading to reduced replacement costs and increased system availability [4]. Additionally, they ensure high performance and improved quality of the generated energy due to their ability to generate an output signal with lower harmonic content, reducing disturbances in the electrical grid and enhancing system efficiency.

The designed hardware in this paper has one of its main objectives to serve as an experimental platform for studying configurations of multilevel converters and the control algorithms applied to them, focused on their application in renewable energy-based generation systems, especially wind and photovoltaic solar systems. A modular and scalable design was implemented to achieve the latter, allowing it to adapt to different working configurations required for the experiments. The main element of the proposed converter design is the individual H-bridge cells, which form a complete and independent system to provide this platform versatility and scalability. H-bridge cells comprise two boards: the power board and trip controller circuits. From a practical point of view, these devices are interconnected using modular and independent printed circuit boards (PCBs), thus facilitating the assembly and preventive-corrective maintenance of the converter. H-bridge cells can be grouped to form various configurations of power converters based on the H-bridge. As the case study for this article, the configuration of three cells for each phase forms a three-phase converter of seven levels per phase.

The trigger control circuit for the H-bridge receives the pulse width modulation (PWM) trigger signals from the control unit, such as a digital signal processor (DSP), through an optic fibre link to reduce electromagnetic interferences generated by the switching circuits that may alter the correct operation of the controller. The power semiconductors for the power board design are SiC-MOSFET semiconductors mounted on a board that integrates the signal conditioning circuits necessary for its correct operation. The measurement and conditioning circuits for voltages and currents were designed to measure electrical variables necessary for implementing control algorithms. These measurement boards also have a modular structure that allows quick assembly and replacement in case of failures. All the circuits and PCBs mentioned above form an experimental test bench of an electronic power converter to analyse and evaluate different control algorithms and hardware configurations.

## 2 Hardware description

### 2.1 Multilevel Power Converter Integration

**Fig. 1:**
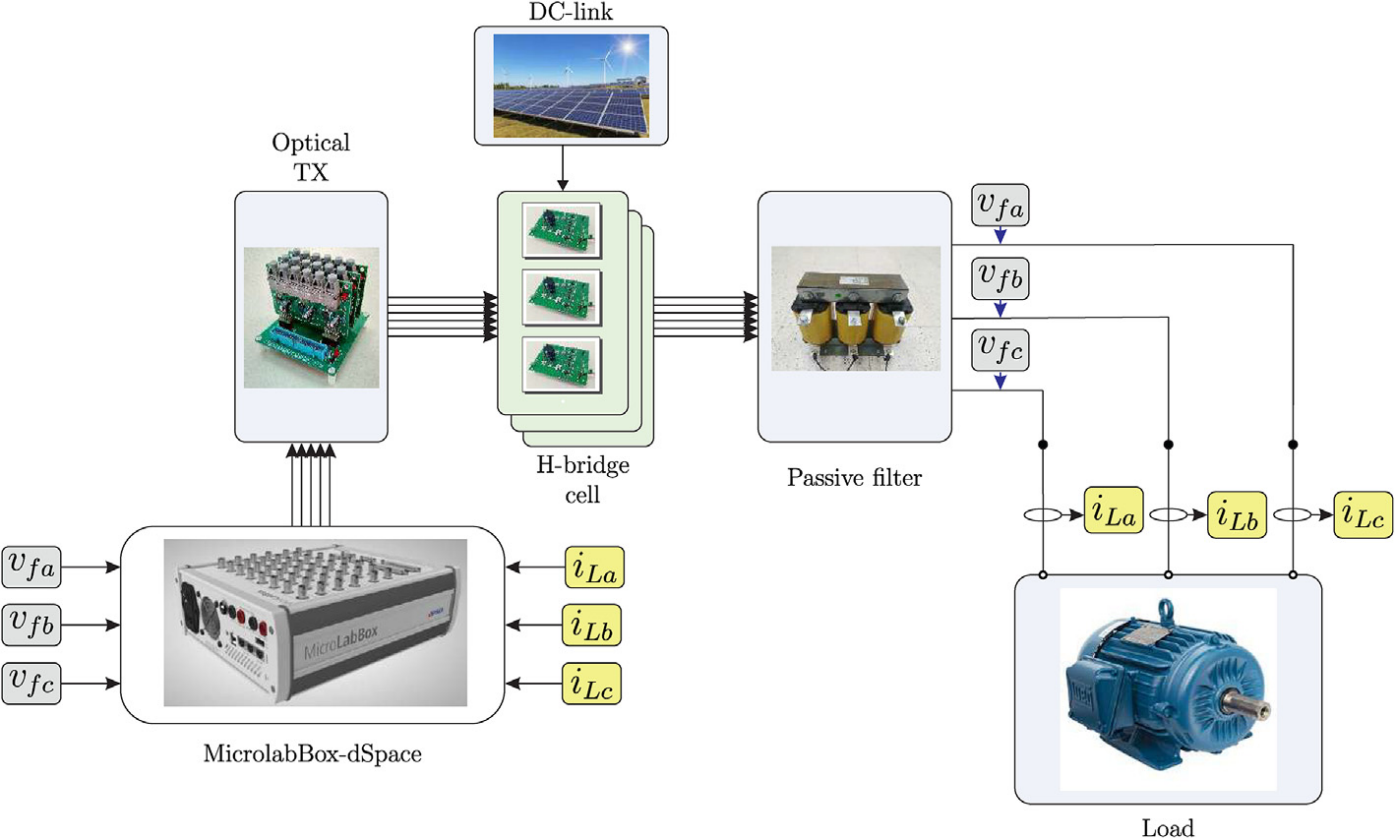
Block diagram of the experimental setup, including the multilevel converter, load, control board and DC source.

Fig. 1 shows the final scheme implemented in the design of the multilevel converter. The system consists of a MicroLabBox dSPACE controller device for implementing different control algorithms. This development device makes it possible to perform experimental tests and/or measurements quickly and easily, mainly due to its programming environment based on MatLab/Simulink software and its native application for developing SCADA-type graphical interfaces. DSP’s high computational power and low I/O latencies provide good real-time performance [5].

The MicroLabBox dSPACE interacts with the physical system of the experimental test bench through measurement and signal conditioning circuits, obtaining the necessary variables for the control algorithm, which are the load currents and the converter’s output voltage. Once the external information is obtained, the control algorithm calculates the switching vectors for each H-bridge cell, sending trigger signals through the GPIO outputs of the controller, which operate at TTL levels (5 V). Finally, the trigger signals are transmitted through an optic fibre link to the SiC-MOSFET trigger control board, integrated into the power board with signal conditioning circuits and noise levels reduction generated by the semiconductor switching.

### 2.2 H-bridge cell design based on SiC-MOSFET

**Fig. 2:**
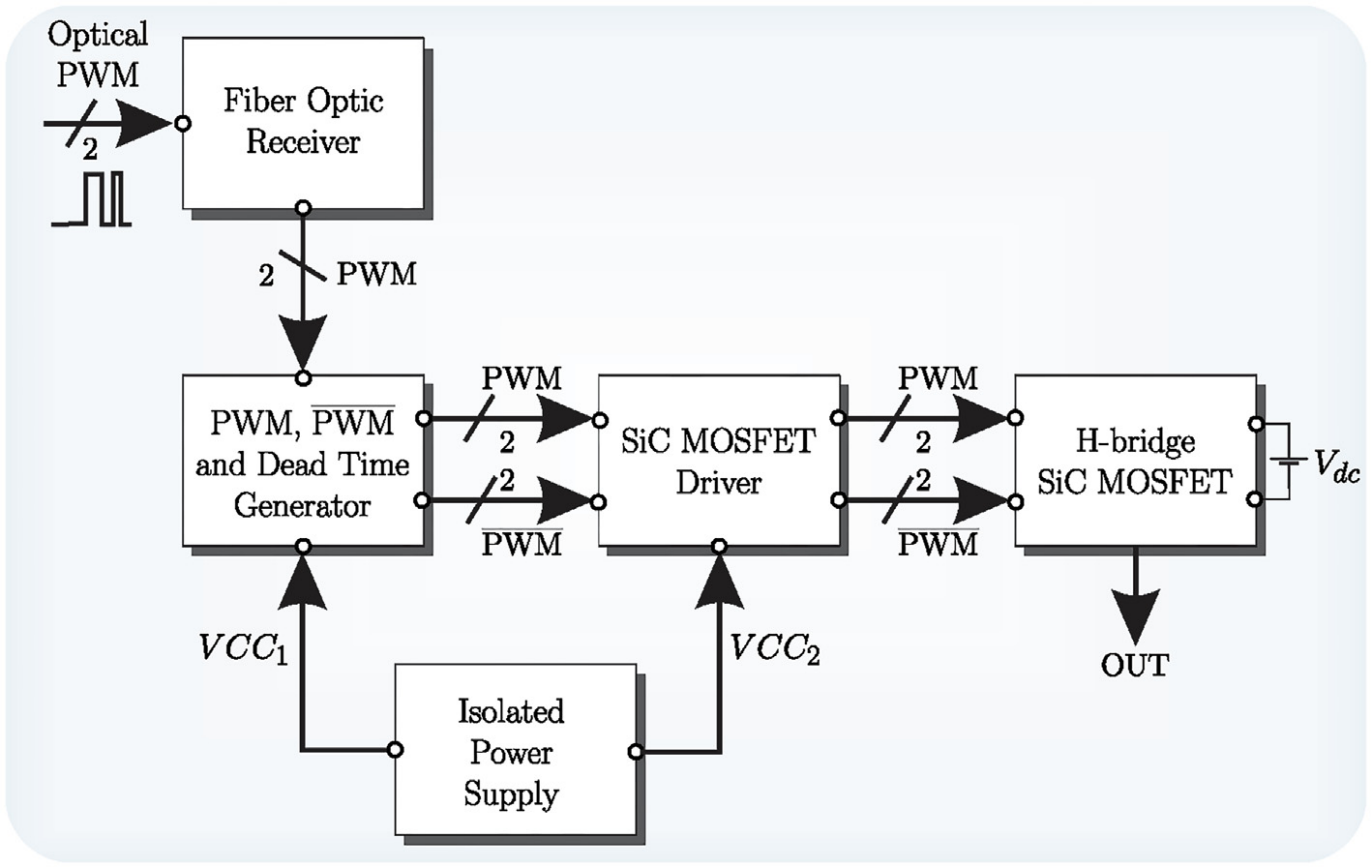
H-bridge controller and power board block diagram.

The block diagram of the proposed H-bridge cell with the power board is shown in Fig. 2. The control signals are received via an optic fibre link to reduce electrical noise levels in the digital control circuits and measurement systems, using the HFBR-2521Z optic fibre receiver [6]. Subsequently, the received PWM signals are sent to the block, generating the necessary complementary trigger signals for each branch of the H-bridge. This logic circuit implements the dead time between a PWM signal and its complement. It is achieved using 74HC86 XOR gates and resistive and capacitive elements to generate the dead time delays. The driver for the SiC-MOSFETs is implemented using the IR2110S integrated circuit. The SiC-MOSFET CAS120M12BM2 is used to assemble the design power stage. Their independent power supply is implemented by DC/DC converter CC10-1212SF with galvanic isolation to achieve modularity of each H-bridge cell.

#### 2.2.1 Trigger control circuit for H-bridge

**Fig. 3:**
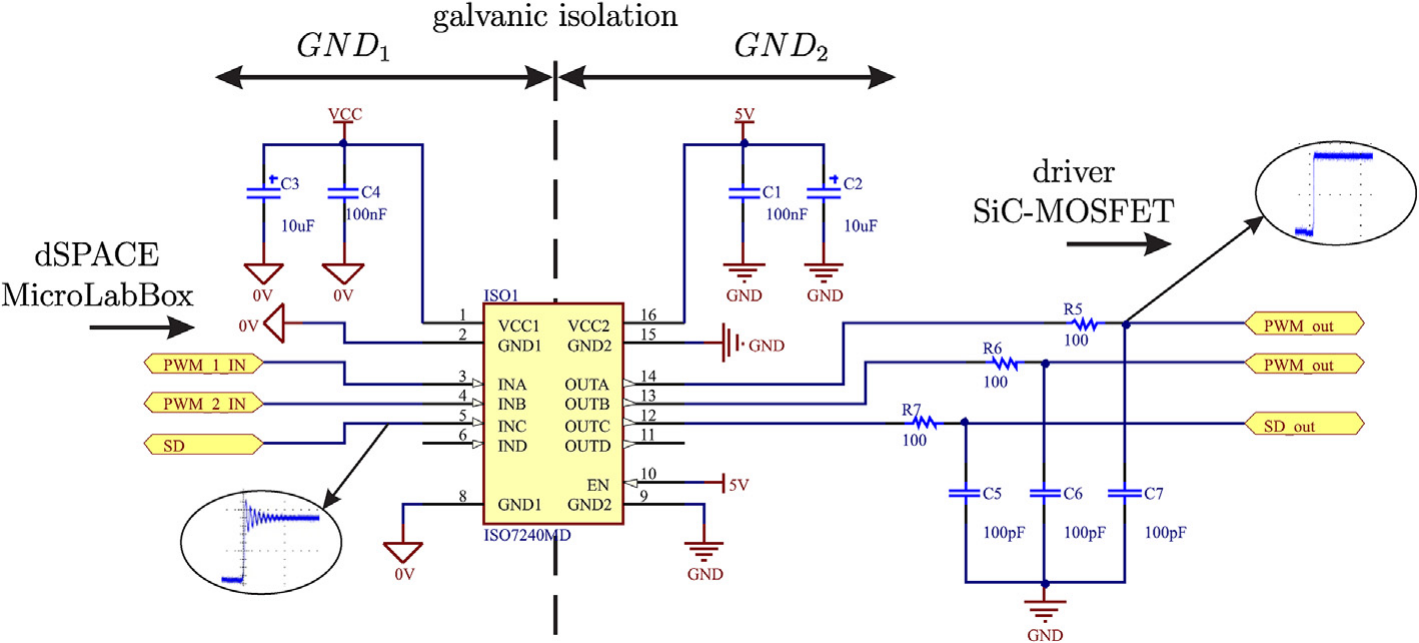
PWM signal conditioning circuit.

**PWM signal conditioning**:

The PWM signals transmitted from the dSPACE control device must be conditioned to eliminate or attenuate the presence of oscillations or overshoot during transitions between voltage levels at high frequencies. Signal conditioning is implemented using the ISO7240 integrated circuit, which provides galvanic isolation up to 2500 V_*RMS*_ for 1 min [7]. These devices allow high voltage blocking, isolate grounds, and prevent noise currents from entering the local ground, interfering with or damaging sensitive circuits. Fig. 3 shows the design based on ISO7240, according to design guidelines provided by the manufacturer [8].

The RC filter used at the output of each channel of the ISO7240 is used to reduce the overshoot during transitions between logic levels. It is considered that the maximum switching frequency will be 100 kHz. Therefore, it is chosen to size the filter with a cut-off frequency higher than the maximum operating frequency in such a way that it generates significant phase shifts. The values used for the low pass filter are R  = 100 Ω and C  = 100 pF, obtaining an approximate cut-off frequency of 1.59 MHz.

**Fig. 4:**
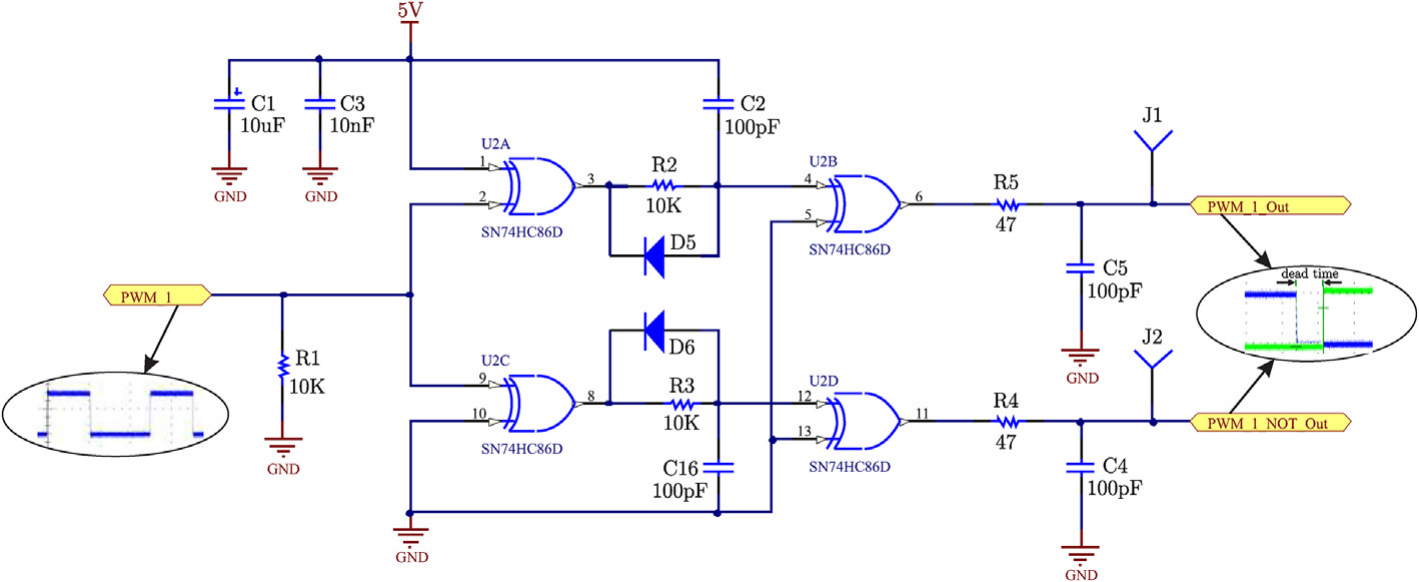
Dead time and complementary signal generating circuit.

**Complementary PWM Signal Generation and Dead Time**:

In real applications, any signal transition can be considered as a very fast falling or rising ramp [9] instead of perfect transitions, which would cause damage to the power devices due to current overshoot caused by small short circuits during the rising or falling transitions of PWM signals. The circuit in charge of generating the complementary signals and dead times is shown in Fig. 4. The implementation contemplates a similar scheme for each required PWM channel.

Dead time is determined according to the characteristics of trigger driver ICs for SiC-MOSFETs. This is mainly due to the delays generated by on ($t_{\mathrm{on}}$) and off ($t_{\mathrm{off}}$) times. The manufacturer’s technical data sheet of the gate driver device manufacturer for the SiC-MOSFET indicates these times $t_{\mathrm{on}}$ = 120 ns and $t_{\mathrm{off}}$ = 120 ns. The SiC-MOSFET used is the CAS1201012BM2, it has these times $t_{\mathrm{on}}$ = 38 ns and $t_{\mathrm{off}}$ = 70 ns, therefore a dead time $t_{d}$ = 1 μs has been implemented. The assigned value to the dead time is set by elements R2-C2 and R3-C16 as seen in Fig. 4, diodes D5 and D6 should be of the fast switching type. PWM signals, together with their complement, pass through an Low pass filter (LPF) formed by R5-C5 and R4-C4 to reduce the existing over-peaks or ripples at the output of the stage without adding a significant phase shift to these signals.

**Fig. 5:**
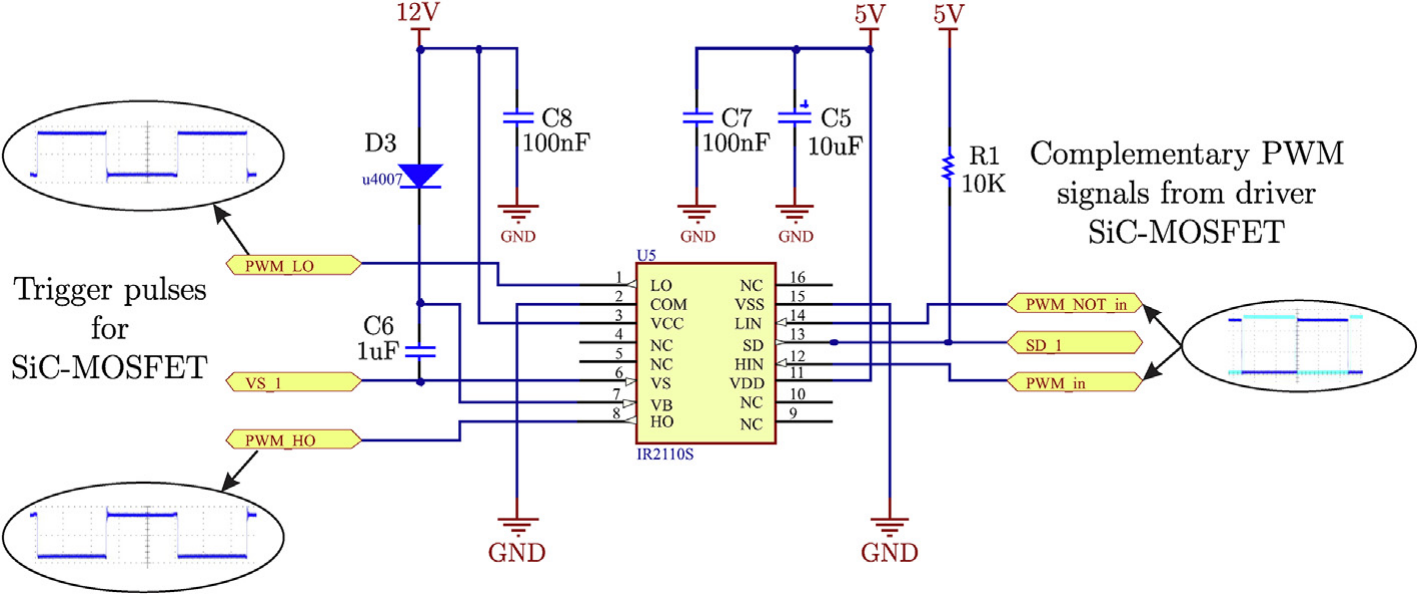
Electrical diagram of the circuit implemented with IR2110.

**Trigger control for SiC-MOSFET**:

A design based on commercial integrated circuits for H-bridge control is developed for the switching control of the power semiconductors of the H-bridge cells. The IR2110S integrated circuit of the International Rectifier company has been used to implement this design [10]. The IR2110S is a high-speed MOSFET and IGBT driver with independent high and low trigger channels. The logic inputs are compatible with standard CMOS and TTL levels. Typical wiring of the IR2110S integrated circuit, according to the manufacturer’s technical specifications and according to the proposed design criteria, is shown in Fig. 5.

The IR210 integrated circuit manufacturer’s data sheet recommends using a capacitor and a diode ($C_{6}$ and $D_{3}$) to implement the Bootstrap triggering technique, as shown in Fig. 5. Using (1), it is possible to calculate the value of the Bootstrap capacitor according to the manufacturer’s technical data.

(1) $$C_{\mathrm{bs}}⩾\frac{2*\langle 2*Q_{g}+\frac{I_{\mathrm{qbs}(\max)}}{f}+Q_{\mathrm{ls}}+\frac{I_{\mathrm{cbs}(\mathrm{leak})}}{f}\rangle }{V_{\mathrm{CC}}-V_{f}-V_{\mathrm{LS}}-V_{\min}}$$

where $Q_{g}$ is the total gate terminal load, $Q_{\mathrm{ls}}$ the required load level change per cycle, $V_{\mathrm{LS}}$ is the voltage drop across the bottom SiC-MOSFET, $V_{f}$ the voltage drop across the direct biased Bootstrap diode and $I_{\mathrm{qbs}(\max)}$ is the maximum quiescent current value for the high side of the driver (upper SiC-MOSFET). The values used are shown in Table 1.

**Table 1:** Electrical parameters used to calculate the Bootstrap.

Parameter	Value	Unit
Supply voltage VCC	12	V
Operating frequency	100	kHz
$Q_{g}$	15	nC (Typ.)
$Q_{\mathrm{ls}}$	5	nC
$V_{\mathrm{LS}}$	10	V
$V_{f}$	1	V
$I_{\mathrm{qbs}(\max)}$	230	A

For the calculations, the term $\frac{I_{\mathrm{cbs}(\mathrm{leak})}}{f}$ and $V_{\min}$ are disregarded because the capacitors are not electrolytic. The final value resulting from the calculations for the $C_{\mathrm{bs}}$ capacitor is 74.6 nF, for which 100 nF has been used as the final value.

**Fig. 6:**
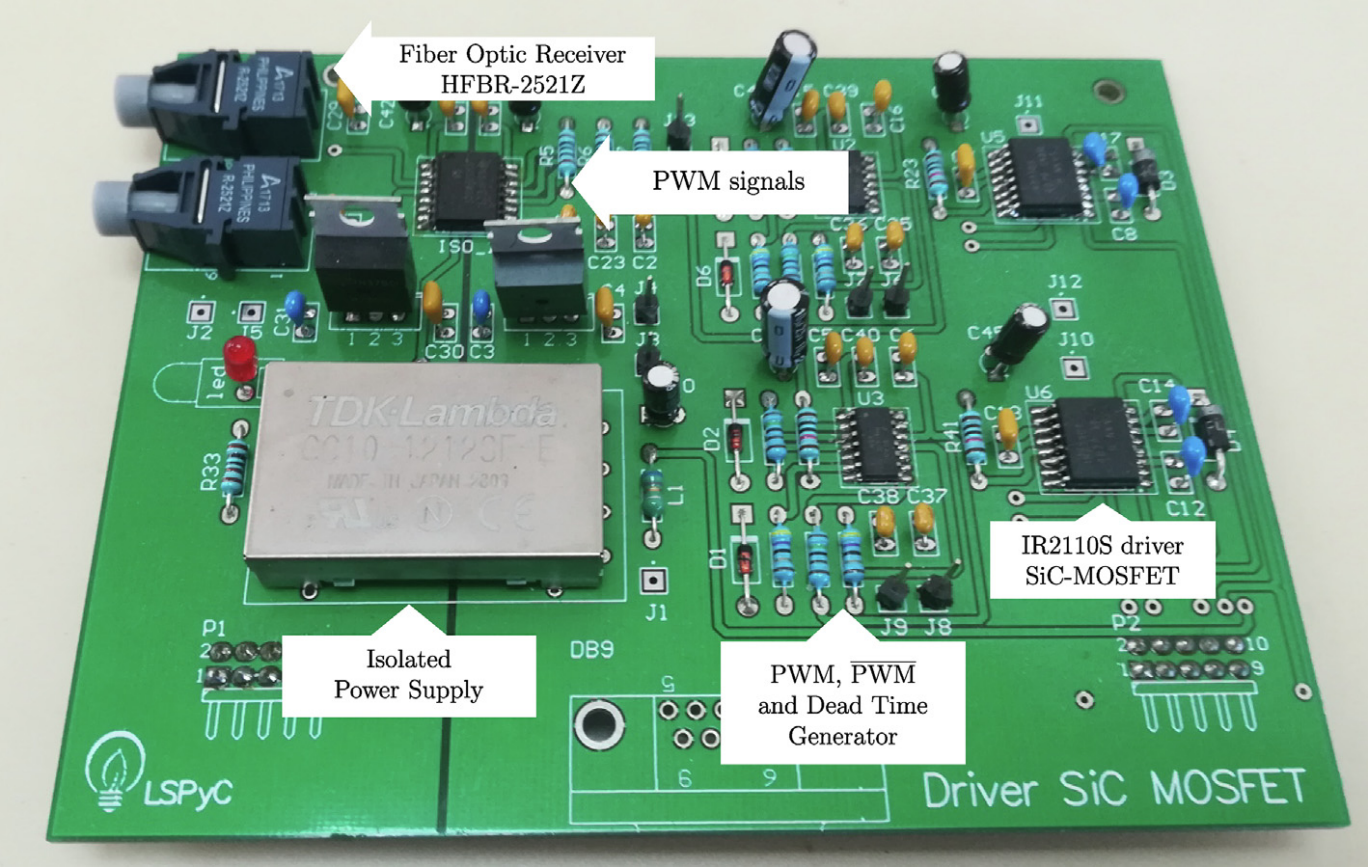
Control board PCB for H-bridge cells based on SiC-MOSFET.

**PCB board designed for the trigger control circuit**:

Fig. 6 shows the final PCB resulting from the detailed design in previous sections. The image shows the most representative parts of the PCB.

### 2.3 Design of the power circuit for the H-bridge

The proposed solution for designing the multilevel converter is the integration of cells in an H-bridge configuration connected in series to obtain higher output voltage levels. This latter allows for meeting the objective of a scalable and modular design. Table 2 shows the most relevant design criteria that are taken into account for the power stage.

**Table 2:** Design criteria for the power board.

Parameter	Value	Unit
DC-link voltage	400	V
Maximum power	1000	W
Operating frequency	50	kHz

To achieve this objective, the DC-link must be implemented through a floating voltage, i.e., not referenced to GND, and simultaneously independent in each H-bridge cell. The power stage allows optional implementation of DC-link by using external DC power supplies for each DC-link voltage.

**DC-link voltage calculation**:

The maximum DC-link voltage for each H-bridge cell must be such that it can be implemented in various multilevel configurations, operating with a significant safety margin. For this converter design, calculations were performed considering a maximum DC-link voltage for each cell of 400 V.

In addition, if the DC-link voltage is 400 V, it is possible to obtain an output voltage per phase of 1200 V with three H-bridge cells, thus, it is possible to operate with a large safety margin in 220/380 V grids. The value of the capacitor for the DC-link can be expressed by (2).

(2) $$C=\frac{P_{\mathrm{nom}}}{2*\omega *V_{\mathrm{DC}}*\Delta V_{\mathrm{DC}}}$$

being the power value P_*nom*_ = 1000 W, the voltage over the capacitor $V_{\mathrm{DC}}$ = 400 V, with a variation of 1% in $\Delta V_{\mathrm{DC}}$ = 4 V, resulting in a minimum value of C  = 497 μF.

**Power semiconductor devices**:

The SiC-MOSFET CAS120M12BM2 from CREE Semiconductor is a two-transistor power module with a breakdown voltage of 1200 V for a current value of 138 A for a 90°C temperature and a low conduction resistance with a high switching speed, higher than traditional switching devices such as IGBTs.

Table 3 shows the most relevant parameters of the selected SiC-MOSFET [11]. Then, to avoid damage to the H-bridge cell due to voltage transients that may occur, a safety margin in voltage and current levels of 80% is proposed for the selection of the switching devices. Therefore, the levels to be withstood by the SiC-MOSFETs are 720 V and 15 A, respectively. Furthermore, it is observed that the selected device, the CAS120M12BM2 meets the maximum voltage, maximum current, power dissipation and speed requirements demanded by the proposed design for the multilevel converter.

**Table 3:** Electrical parameters of the SiC-MOSFET CAS120M12BM2 at 25°C.

Parameter	Symbol	Value	Unit
Drain-Source Voltage	VDSmax	1.2	kV
Gate-Source Voltage	VGSmax	−10/+25	V
Drain current in continuous mode	ID	193	A
Diode reverse current	IF	305	A
Junction temperature	TJmax	−40 to + 150	°C
Power dissipation	PD	925	W
Resistance ON mode	RDS(on)	13–16	mΩ

**Snubber network design for the switches**:

Electronic switches are the fundamental element of all power converters. To obtain high performance of the converter switching device, it is necessary to design damping networks, also known as snubber networks, to reduce phenomena such as voltage surges and transient damping caused by circuit inductances when switching a transistor. These phenomena can exceed the devices’ physical limits, resulting in the degradation or destruction of such devices. These transients can be detrimental to electronic devices and can adversely affect system efficiency and reliability. A snubber network provides an alternative path for the current and absorbs the energy stored during switching [12].

**Fig. 7:**
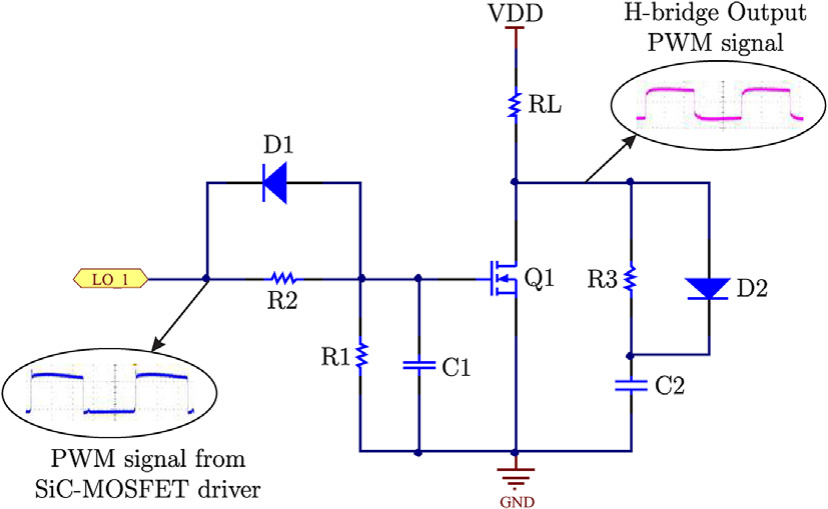
Electrical schematic of RCD type snubber network.

The selected snubber network for the power board design is the RCD-type network. This circuit topology finds a wide field of application in the protection of power switches, such as bipolar transistors or IGBTs, due to its simplicity and efficiency [13]. The schematic representation of the selected snubber network is shown in Fig. 7.

The value of the required capacitance for the snubber network is determined by the following Eq. (3), which determines the minimum value required for the design conditions.

(3) $$C_{s}=\frac{L_{M}*I_{\mathrm{off}}^{2}}{{\left(V_{\mathrm{DSP}2}-V_{\mathrm{DD}}\right)}^{2}}$$

where $L_{M}$ is the parasite inductance value, $V_{\mathrm{DSP}2}$ corresponds to the DC peak voltage over the capacitor $C_{s},I_{\mathrm{off}}$ is the off current and $V_{\mathrm{DD}}$ is the DC-link voltage value. The following values were used: $L_{M}$ = 15 nH, $V_{\mathrm{DSP}2}$ = 480 V, $V_{\mathrm{DD}}$ = 400 V y $I_{\mathrm{off}}$ = 8 A to determine the capacitor value $C_{s}$, resulting in an approximate value of $C_{s}$ = 150 pF. To calculate the resistance value $R_{s}$ we used Eq. (4).

(4) $$R_{s}⩽\frac{1}{2.3*C_{s}*\mathrm{fs}}$$

Considering the maximum switching frequency of $f_{s}$ = 100 kHz, and assuming the value of $C_{s}$ previously obtained by 3, the approximate value of $R_{s}$ = 28.9 kΩ is obtained. The connected diode to the following elements, $R_{s}$ y $C_{s}$, has to be of fast switching type and capable of operating in the current ranges of the H-bridge scheme. The selected component is the ST2045A diode, a Schottky diode capable of driving an average current of up to 20 A and a maximum peak current of 250 A. A network made up of a diode, and a resistor is added to the snubber input at the gate terminal of the SiC-MOSFET, as shown in Fig. 7.

This arrangement generates a small activation delay without affecting the SiC-MOSFET turn-off, providing additional dead time. The resistor-diode network is used to reduce the overshoot that exists during the reverse recovery time of the device. On the other hand, the IR2110S controller can deliver a current of up to 2 A at its outputs during the trip. This allows limiting the value of said current delivered at the gate terminal of the SiC-MOSFET. For this, a resistor is used $R_{g}$ = 47 Ω, being the trigger voltage at the output of the gate terminal $V_{\mathrm{gs}}$ = 12 V, resulting in an output current of $I_{o}$ = 250 mA for that voltage.

An RC low-pass filter is implemented in the input circuit, which reduces the overshoots and oscillations of the signals coming from the IR2110 controller. The RC filter capacitor results from the parasitic capacitance of the SiC-MOSFET, which has a value of $C_{\mathrm{iss}}$=6,3 nF according to the manufacturer’s manual, while the external capacitor used for the filter has a value of 7.8 nF. The resistor connected to the gate terminal of the device has a value of $R_{g}$ = 47 Ω, resulting in a cutoff frequency equal to $f_{0}$ = 434 kHz.

**Fig. 8:**
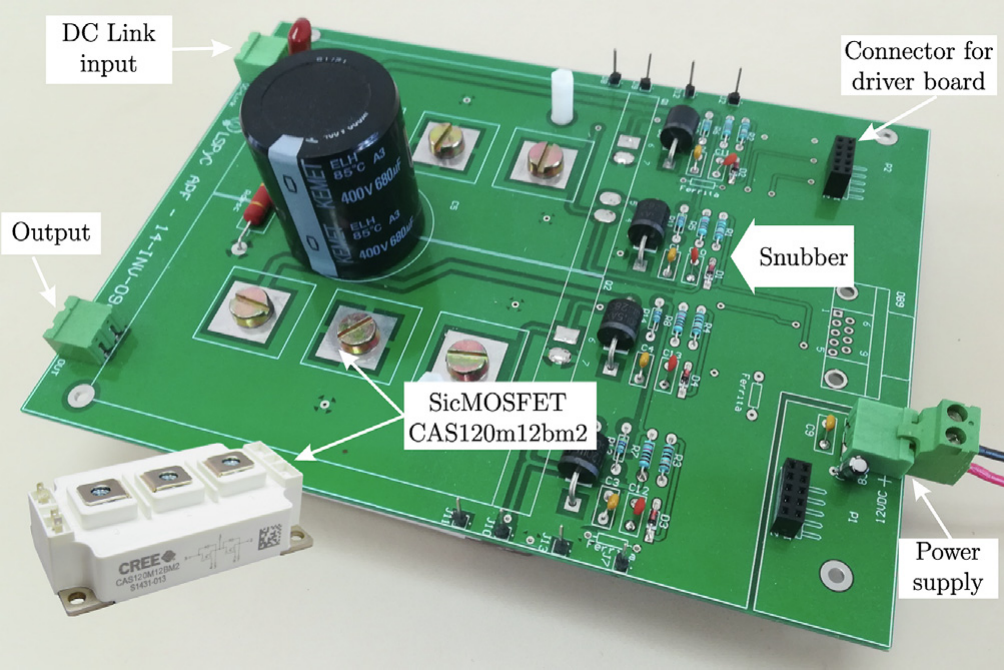
Implementation of the power board for an H-bridge cell based on SiC-MOSFET device.

**PCB board resulting from the design of the power circuit**:

Fig. 8 shows the final PCB of the H-bridge cell power board, resulting from the design mentioned in previous sections, where the most representative blocks are highlighted, as well as the power semiconductor used for the H-bridge. The board has connectors to connect to the trigger controller board for the SiC-MOSFETs, as well as mounting points to provide mechanical rigidity to the trigger controller board/power board assembly.

**Fig. 9:**
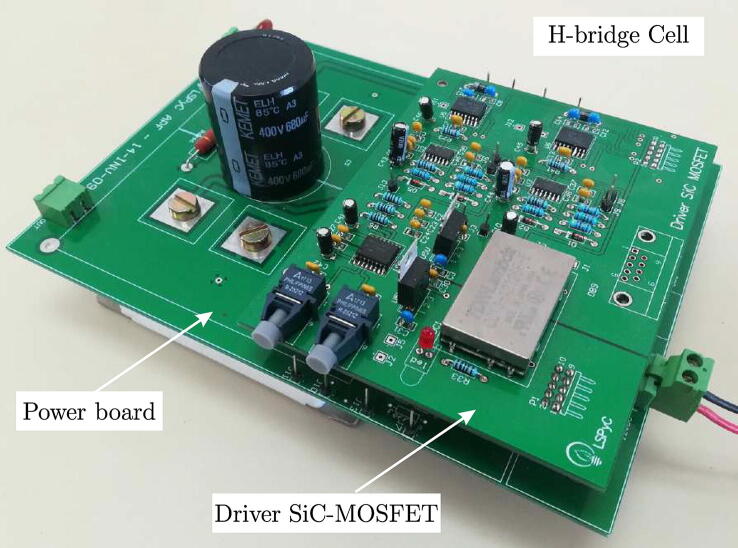
Assembly of the H-bridge cell.

**Final assembly of the H-bridge cell**:

Fig. 9 shows the final assembly of the H-bridge cell, made up of the power board and the trigger controller board. These boards are interconnected through two 5x2 header-type connectors, one of the connectors for signal transfer and the second for supply voltages.

### 2.4 Voltage Signal Conditioning

**Fig. 10:**
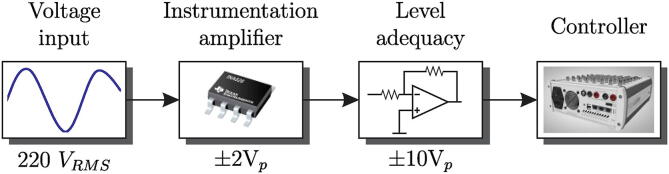
Diagram of the voltage measurement process.

To implement the control algorithms, it is necessary to carry out the measurement and conditioning process of the signals corresponding to the relevant electrical variables for the various algorithms. The scheme of the acquisition voltage signals is shown in Fig. 10.

**Fig. 11:**
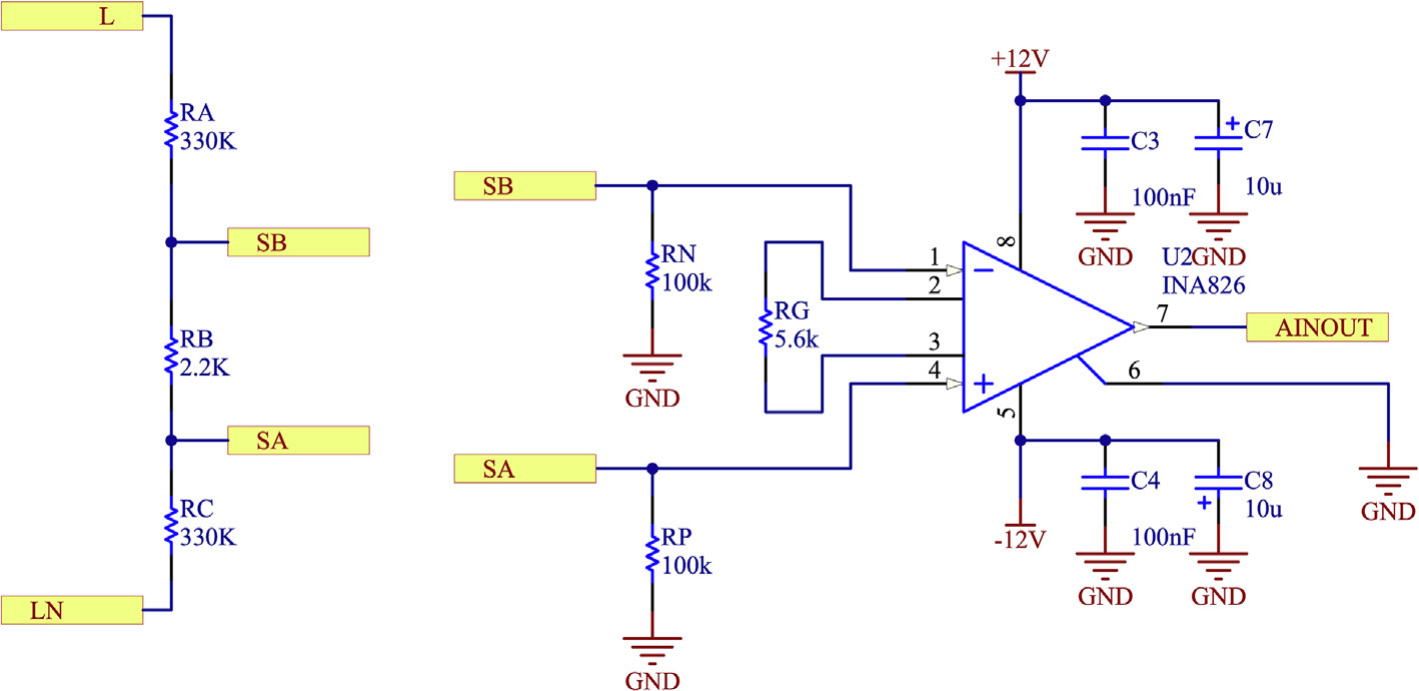
Schematic of step-down stage of voltage input level.

In the voltage measurement circuit, an initial stage consisting of a resistive voltage divider is used to connect the electrical network signal to be processed. The resistive divider is necessary to reduce the voltage amplitude to manageable levels by the INA826 low noise instrumentation amplifier used for the proposed design [14]. The scheme for the input stage of the voltage sensor is observed in Fig. 11.

The input voltage divider is designed for a maximum input voltage of 380 Vrms, equivalent to 537.39 Vp. The maximum input level is set to 600 Vp. The input voltage divider provides 0.003323 attenuations to the measured voltage, resulting in a voltage of 1.9938 Vp at the input of the INA826. The gain of the INA826 instrumentation amplifier is given by (5):

(5) $$G=1+\frac{49.4k\Omega }{\mathrm{RG}}$$

The selected RG value is RG=5.6 kΩ, with which a profit of 9.82 units is obtained.

**Fig. 12:**
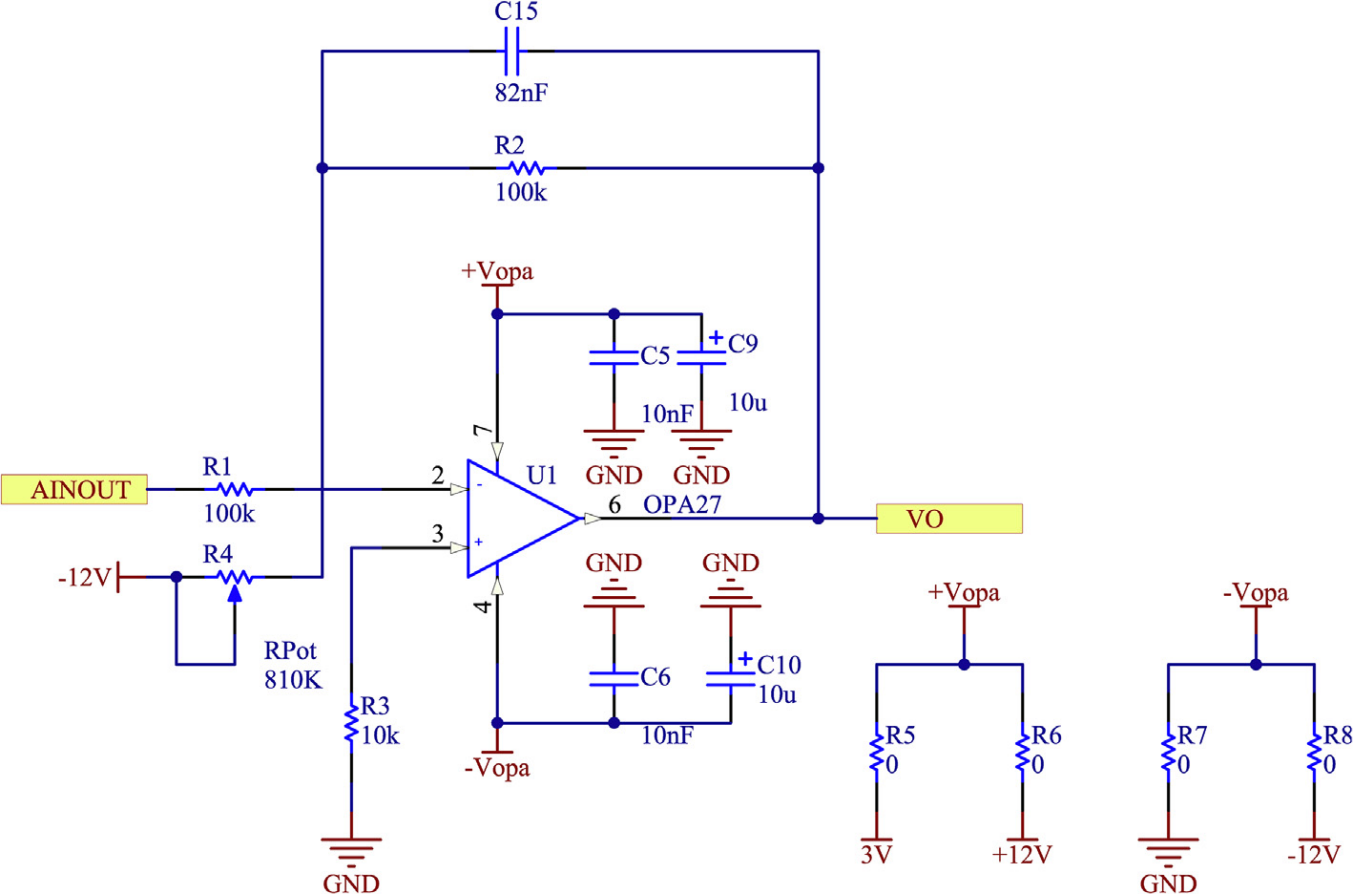
Schematic of offset adjustment stage.

Subsequently, the obtained signal at the output of the INA826 is taken to the second amplifier stage, made up of an ultra-low noise operational amplifier of the OPA27/OPA350 series [15], [16], which is also responsible for modifying the DC offset level and providing a second amplification of the conditioned signal. Fig. 12 shows the schematic of the measurement and conditioning circuit for voltage signals.

**Fig. 13:**
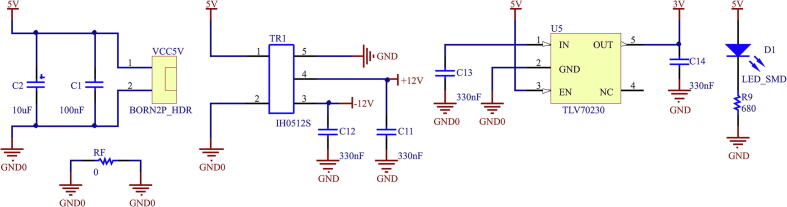
Schematic of DC/DC supply isolated from voltage sensor.

The printed circuit design allows the voltage meter to be adapted to output levels of ± 10 V and 0–3 V, respectively. This is possible by modifying the second amplifier stage, selecting the OPA27 for the ± 10 V case or the OPA350 for the 0–3 V case. In addition, when selecting the operational amplifier for this stage, the supply voltages must be modified according to the case. This is achieved by modifying R5-R6 for the positive supply and R7-R8 for the negative supply. This stage is configured to obtain unity gain, being able to modify the offset level by using preset R4. In addition, the necessary capacitive filters are implemented to provide a good response to power interference. The voltage sensor board implements a galvanically isolated power supply using an IH0512S galvanic isolation switched DC/DC supply [17]. Fig. 13 shows the schematic circuit of the isolated source.

Using IH0512S, the voltages ± 12 V are set, from which the other necessary voltages are obtained in the case of requiring the use of the OPA350 for a 0–3 V output. This is achieved through the use of 3 V voltage regulators.

**Fig. 14:**
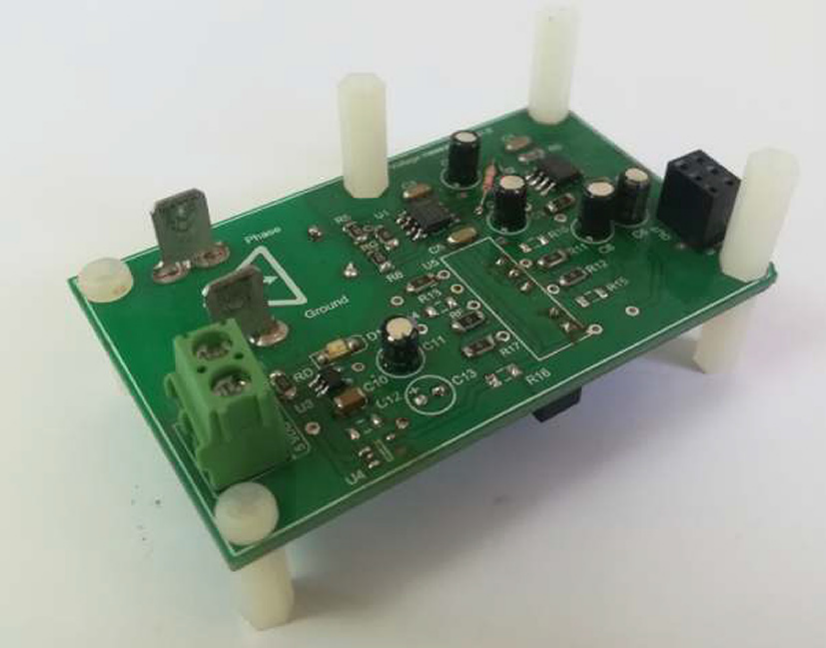
Voltage measurement and conditioning board.

**PCB board resulting from the voltage sensor design**:

The implementation of the voltage measurement and conditioning circuits on modular printed circuit boards can be seen in Fig. 14.

### 2.5 Current signal conditioning

**Fig. 15:**
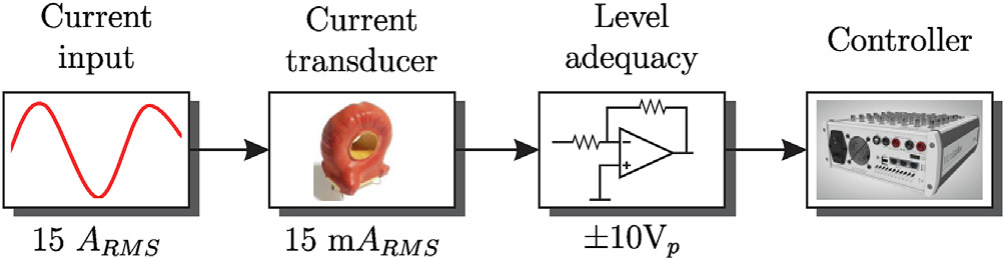
Diagram of the current measurement process.

The circuit diagram for the measurement and conditioning of current signals is shown in Fig. 15.

**Fig. 16:**
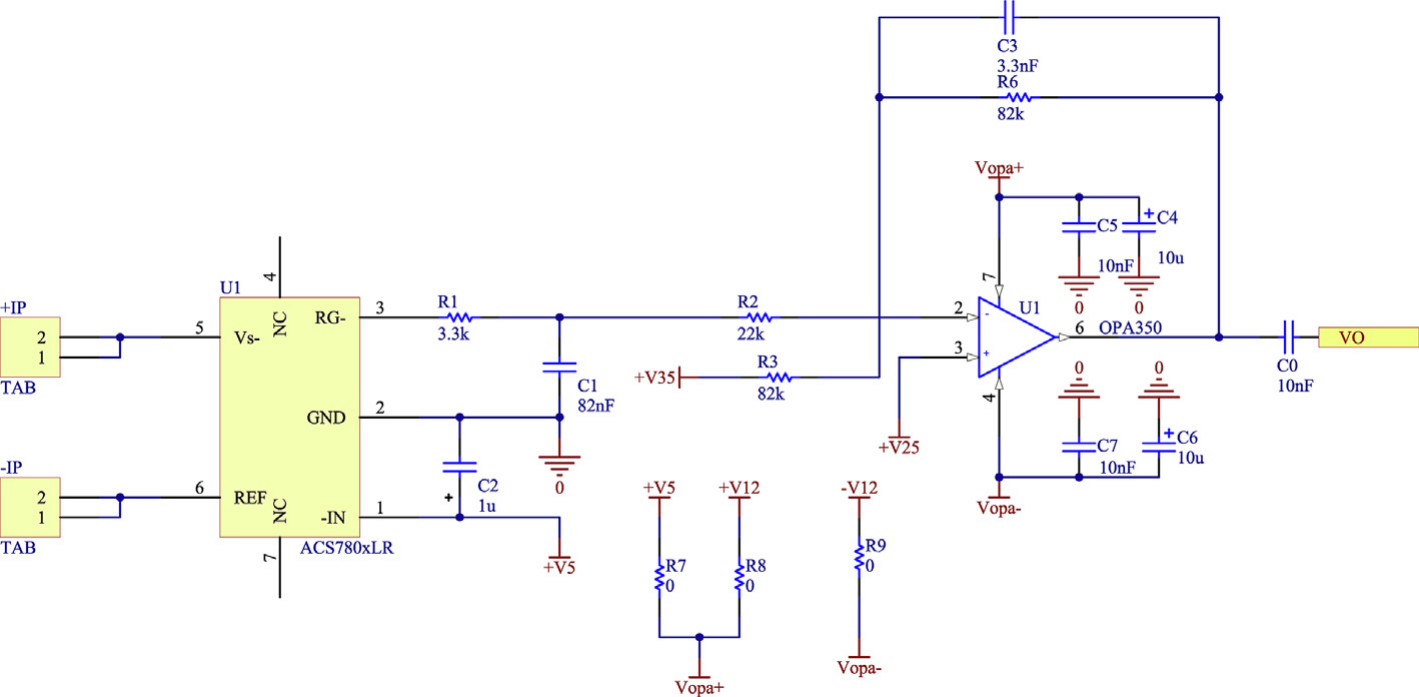
Schematic of the step-down stage of the input level for current.

The first stage implements the ACS780xLR sensor [18], a current sensor integrated circuit designed to measure AC and DC currents up to 100 A. The ACS780xLR sensor, for a maximum input range of ±50 A, has a sensitivity of 40 mV/A according to its datasheet, resulting in a maximum output value of 2 V. Subsequently, the output signal obtained from the ACS780xLR sensor is sent to the amplifier and offset level adjustment stage, which stage is configured with unity gain. The design of the printed circuit allows adapting the output voltage level to output levels of ± 10 V and 0–3 V, respectively. This is possible by modifying the second amplifier stage, selecting the OPA27 for the ± 10 V case or the OPA350 for the 0–3 V case, just like the voltage sensor circuit. To select the OPA27, use R8 and R9, while the OPA350 uses R7. The integrated circuits have capacitive filters to reduce the levels of electrical noise in their feeds. Fig. 16 shows the schematic of the circuit corresponding to the current input level reduction stage.

**Fig. 17:**
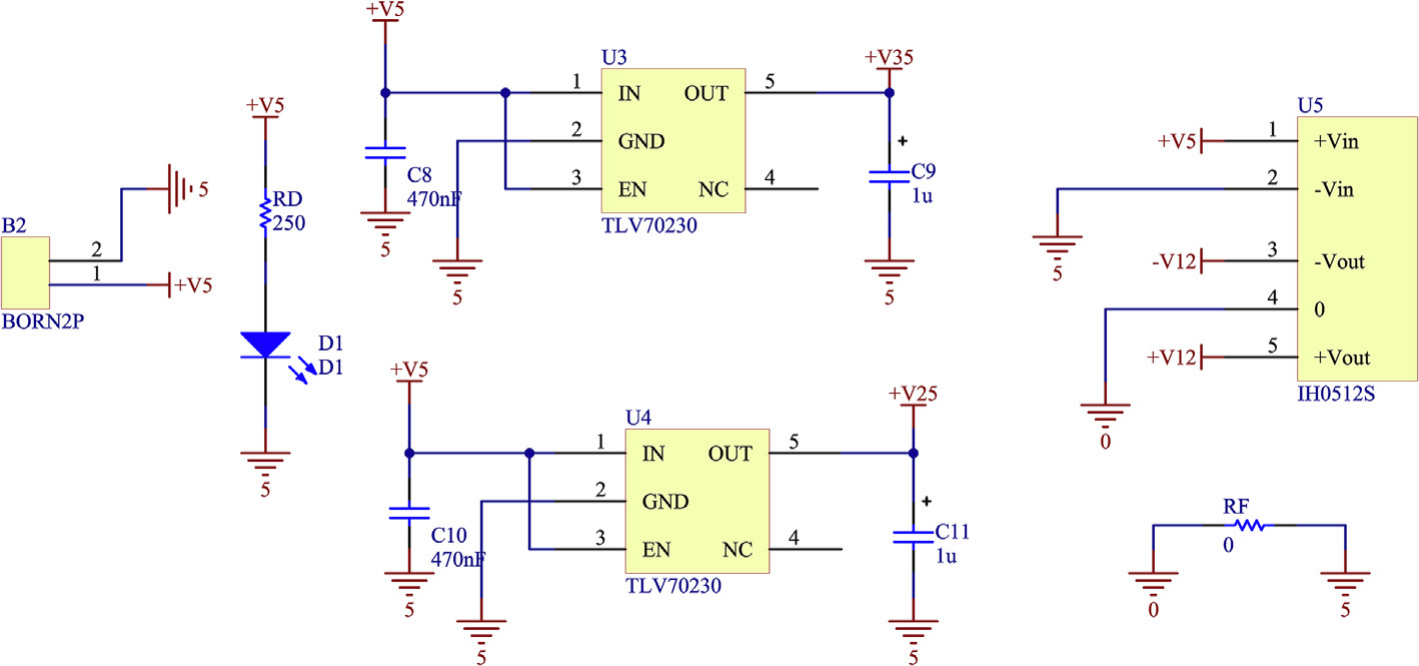
Isolated DC/DC power schematic from current sensor.

The current sensor board uses a galvanically isolated power supply through the IH0512S switched DC/DC power supply, from which the voltages ± 12 V are obtained, and the other voltages are also obtained, in case of the use of the OPA350 for a 0–3 V output. This is achieved through the use of 3 V voltage regulators. Fig. 17 shows the schematic of the circuit that implements the board’s power supply.

**PCB board resulting from current sensor design:**

**Fig. 18:**
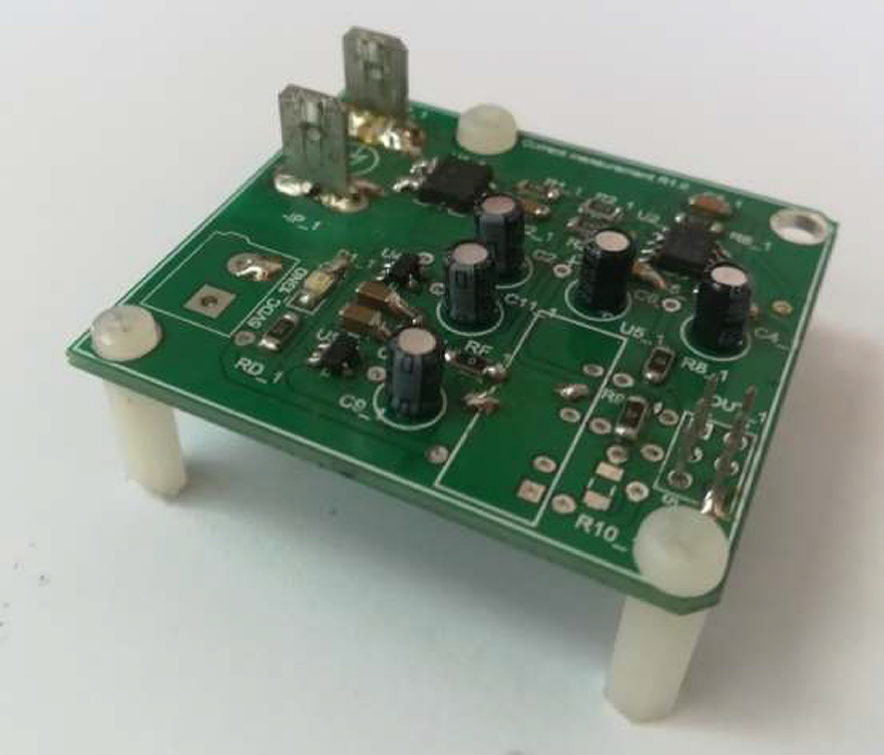
Current measurement and conditioning board.

The implementation of the current measurement and conditioning circuits on modular printed circuit boards can be seen in Fig. 18.

## 3 Design files summary

.

**Table:** 

**Design filename**	**File type**	**Open source license**	**Location of the file**
*Current Sensor*	*PCB file and Gerber, Altium*	*GNU GPL v3*	*https://osf.io/95y76*
*currentSenSch.pdf*	*Current Sensor schematic file, pdf*	*GNU GPL v3*	*https://osf.io/zwun9*
*H-bridgeList.pdf*	*List of Components and prices file, xlsx*	*GNU GPL v3*	*https://osf.io/euwq5*
*Driver SiC-MOSFET board*	*PCB file and Gerber, Altium*	*GNU GPL v3*	*https://osf.io/k28te*
*driverSicMos.pdf*	*driver H-bridge schematic file, pdf*	*GNU GPL v3*	*https://osf.io/a34fm*
*Power board*	*PCB file and Gerber, Altium*	*GNU GPL v3*	*https://osf.io/wmp52*
*PowerBoardSch.pdf*	*PowerBoard schematic file, pdf*	*GNU GPL v3*	*https://osf.io/362yg*
*Software*	*Software file, C Code for dSpace*	*GNU GPL v3*	*https://osf.io/8qnvg*
*Voltage Sensor*	*PCB file and Gerber, Altium*	*GNU GPL v3*	*https://osf.io/jhpvz*
*voltagesensor.pdf*	*Voltage Sensor schematic file, pdf*	*GNU GPL v3*	*https://osf.io/tvhd7*

• *Current Sensor: Contains the Altium project files for the current sensor PCB and Gerber.*
• *currentSenSch.pdf: Schematic or electronic diagram of current sensor components.*
• *H-bridgeList.pdf: Schematic or electronic diagram of current sensor components.*
• *Driver SiC-MOSFET board: Contains the Altium project files for the Driver SiC-MOSFET PCB and Gerber file to produce the PCB.*
• *driverSicMos.pdf: Schematic or electronic diagram of Driver SiC-MOSFET components.*
• *Power board: Contains the Altium project files for the Power board PCB and the Power board Gerber file to produce the PCB.*
• *PowerBoardSch.pdf: Schematic or electronic diagram of Power board components.*
• *Voltage Sensor: Contains the Altium project files for the voltage sensor PCB and Gerber file to produce the PCB.*
• *voltagesensor.pdf: Schematic or electronic diagram of voltage sensor components.*
• *Software: The algorithm was implemented with the dSpace controller within the Matlab/Simulink environment.*

## 4 Bill of materials summary

**Table:** 

**Driver SiC-MOSFET circuit**						
**Designator**	**Component**	**Number**	**Cost per unit (US**$**)**	**Total cost (US**$**)**	**Source of materials**	**Material type**
C39, C40	10n Capacitors MLCC	2	0.22	0.44	Mouser Electronics	Electrical
C7, C13, C25, C27, C28, C29	100n Capacitors MLCC	6	0.22	1.32.6	Mouser Electronics	Electrical
C3, C4, C30, C31	330n Capacitors MLCC	4	0.22	0.88	Mouser Electronics	Electrical
C2, C5, C6, C15, C16, C23, C24, C31, C36, C37, C38	100p Capacitors MLCC	1	0.22	2.42	Mouser Electronics	Electrical
C10, C41, C42, C43, C44, C45, C46	10u Capacitors THT	7	0.25	1.75	Mouser Electronics	Electrical
C8, C12, C14, C17	1u Capacitors MLCC	4	0.68	2.72	Mouser Electronics	Electrical
L1	4.7μ Core_Ferrite THT	1	0.2	0.2	Mouser Electronics	Electrical
R5, R6, R7	100 Resistor THT	3	0.1	0.33	Mouser Electronics	Electrical
R9, R10, R19, R20, R23, R41	10 k Resistor THT	6	0.1	0.66	Mouser Electronics	Electrical
R31, R32, R34, R35	47 Resistor THT	4	0.1	0.44	Mouser Electronics	Electrical
R33	470 Resistor THT	1	0.1	0.1	Mouser Electronics	Electrical
LED0	Round LED	1	0.24	0.24	Mouser Electronics	Electronic
ISO1	ISO7240 SOIC-16	1	6.08	6.08	Mouser Electronics	Electronic
U2, U3	74HC86 SOIC	2	0.67	1.34	Mouser Electronics	Electronic
U5, U6	IR2110S SOIC	2	3.78	7.56	Mouser Electronics	Electronic
D3, D4	UF4007 Reel	2	0.45	0.9	Mouser Electronics	Electronic
D1, D2, D5, D6	1N4148 DO35	4	0.19	0.76	Mouser Electronics	Electronic
DC1	CC10-1212SF-E	1	32	32	Mouser Electronics	Electronic
FO1, FO2	HFBR-2521Z	2	15.87	31.74	Mouser Electronics	Electronic
U5V, U5V2	LM7805	2	1.96	3.92	Mouser Electronics	Electronic
P1, P2	Headers 10x2	2	1.42	2.84	Mouser Electronics	Electrical
DB9	D-Sub 9	1	3.52	3.52	Mouser Electronics	Electrical

**Table:** 

**H-bridge power board**						
**Designator**	**Component**	**Number**	**Cost per unit (US**$**)**	**Total cost (US**$**)**	**Source of materials**	**Material type**
C1, C2, C3, C4	1n Capacitors MLCC	4	0.22	0.88	Mouser Electronics	Electrical
C10, C11, C12, C13	1.5n Capacitors MLCC	4	0.22	0.88	Mouser Electronics	Electrical
C9	100n Capacitors MLCC	1	0.22	0.22	Mouser Electronics	Electrical
C14	470n 400 V Capacitors MLCC	1	0.45	0.45	Mouser Electronics	Electrical
C8	10u Capacitors THT	1	0.25	0.25	Mouser Electronics	Electrical
C5	680u 400 V Capacitors Snap In	1	20.32	20.32	Mouser Electronics	Electrical
Rdes	120 k 2 W Resistor THT	1	2.05	2.05	Mouser Electronics	Electrical
R1, R2, R3, R4	10 Resistor THT	4	0.1	0.44	Mouser Electronics	Electrical
R5, R6, R7, R8	10 k Resistor THT	4	0.1	0.44	Mouser Electronics	Electrical
R11, R12, R13, R14	47 Resistor THT	4	0.1	0.44	Mouser Electronics	Electrical
FE1, FE3, FE4, FE5	10μ Core_Ferrite THT	4	0.2	0.8	Mouser Electronics	Electrical
D1, D2, D3, D4	1N4148 DO35	4	0.19	0.76	Mouser Electronics	Electronic
D5, D6, D7, D8	DST2045AX	4	2.47	9.88	Mouser Electronics	Electronic
Q1, Q2	CAS120M 12BM2	2	539.89	1079.78	Mouser Electronics	Electronic
P3, P4, P5	Terminal Blocks 2P	3	1.94	5.82	Mouser Electronics	Electrical
DB9	D-Sub 9	1	3.52	3.52	Mouser Electronics	Electrical

**Table:** 

**Voltage measurement circuit**						
**Designator**	**Component**	**Number**	**Cost per unit (US**$**)**	**Total cost (US**$**)**	**Source of materials**	**Material type**
C15	82n Capacitor SMD	1	0.2	0.2	Mouser Electronics	Electronic
C5, C6	10n Capacitor SMD	2	0.2	1	Mouser Electronics	Electronic
C2, C7, C8, C9	10μ Capacitor THT	4	0.15	0.6	Mouser Electronics	Electronic
C11, C12, C13, C14	330n Capacitor SMD	4	0.2	1	Mouser Electronics	Electronic
D1	Led SMD	1	0.45	0.45	Mouser Electronics	Electronic
C1, C3, C4,	100n Capacitor SMD	3	0.2	0.2	Mouser Electronics	Electronic
R1, R2, RN, RP	100 K Resistor SMD	4	3.2	0.588	Mouser Electronics	Electrical
RG	5.6 K Resistor SMD	1	0.147	0.147	Mouser Electronics	Electrical
RA,RC	330 K Resistor SMD	2	1.27	2.54	Mouser Electronics	Electrical
RB	2.2 K Resistor SMD	1	1.27	1.27	Mouser Electronics	Electrical
R3	10 k Resistor SMD	1	0.147	0.147	Mouser Electronics	Electrical
R5, R6, R7, R8	0 Resistor SMD	4	0.147	0.588	Mouser Electronics	Electrical
RF	1 MHz Ferrite Beads	1	0.69	0.69	Mouser Electronics	Electrical
R9	680 Resistor SMD	1	0.147	0.147	Mouser Electronics	Electrical
R4	10 K Potentiometer	1	2.5	2.5	Mouser Electronics	Electrical
U5	TLV70230	1	0.61	0.61	Mouser Electronics	Electronic
U1	OPA27UA/ OPA350 SOIC-8	1	4.09	4.09	Mouser Electronics	Electronic
TR1	IH0512S	1	8.55	8.55	Mouser Electronics	Electronic
U2	INA826	1	3.56	3.56	Mouser Electronics	Electronic
BORN2P	Fixed Terminal Blocks	1	1.94	1.94	Mouser Electronics	Electrical

**Table:** 

**Current measurement circuit**						
**Designator**	**Component**	**Number**	**Cost per unit (US**$**)**	**Total cost (US**$**)**	**Source of materials**	**Material type**
C4, C6	10μ Elect. Capacitors	2	1.8	3.6	Mouser Electronics	Electronic
C2, C9, C11	1μ Elect. Capacitors	3	1.8	5.4	Mouser Electronics	Electronic
C8, C10	470n Capacitor SMD	2	0.2	0.2	Mouser Electronics	Electronic
C1	82n Capacitor SMD	1	0.2	0.2	Mouser Electronics	Electronic
C0, C5, C7	10n Capacitor SMD	3	0.2	0.6	Mouser Electronics	Electronic
C3	3.3n Capacitor SMD	1	0.2	0.2	Mouser Electronics	Electronic
D1	Led SMD	1	0.45	0.45	Mouser Electronics	Electronic
RD	250 Resistor SMD	1	0.147	0.147	Mouser Electronics	Electronic
R1	3.3 k Resistor SMD	1	0.147	0.147	Mouser Electronics	Electrical
R2	22 K Resistor SMD	1	0.147	0.147	Mouser Electronics	Electrical
R3, R6	82 K Resistor SMD	2	0.147	0.294	Mouser Electronics	Electrical
R7, R8, R9	0 Resistor SMD	3	0.441	0.588	Mouser Electronics	Electrical
RF	1 MHz Ferrite Beads	1	0.69	0.69	Mouser Electronics	Electrical
R9	680 Resistor SMD	1	0.147	0.147	Mouser Electronics	Electrical
U5	IH0512S	1	8.55	8.55	Mouser Electronics	Electronic
U2	ACS780	1	8.41	8.241	Mouser Electronics	Electronic
U1	OPA350/ OPA27	1	4.09	4.09	Mouser Electronics	Electronic
U3	TLV70233	1	0.62	0.62	Mouser Electronics	Electronic
U4	TLV70225 SOT-23	1	1.16	1.16	Mouser Electronics	Electronic
BORN2P	Fixed Terminal Blocks	1	1.94	1.94	Mouser Electronics	Electrical
+IP, -IP	Terminals TAB	2	0.21	0.42	Mouser Electronics	Electrical

## 5 Build instructions

**Fig. 19:**
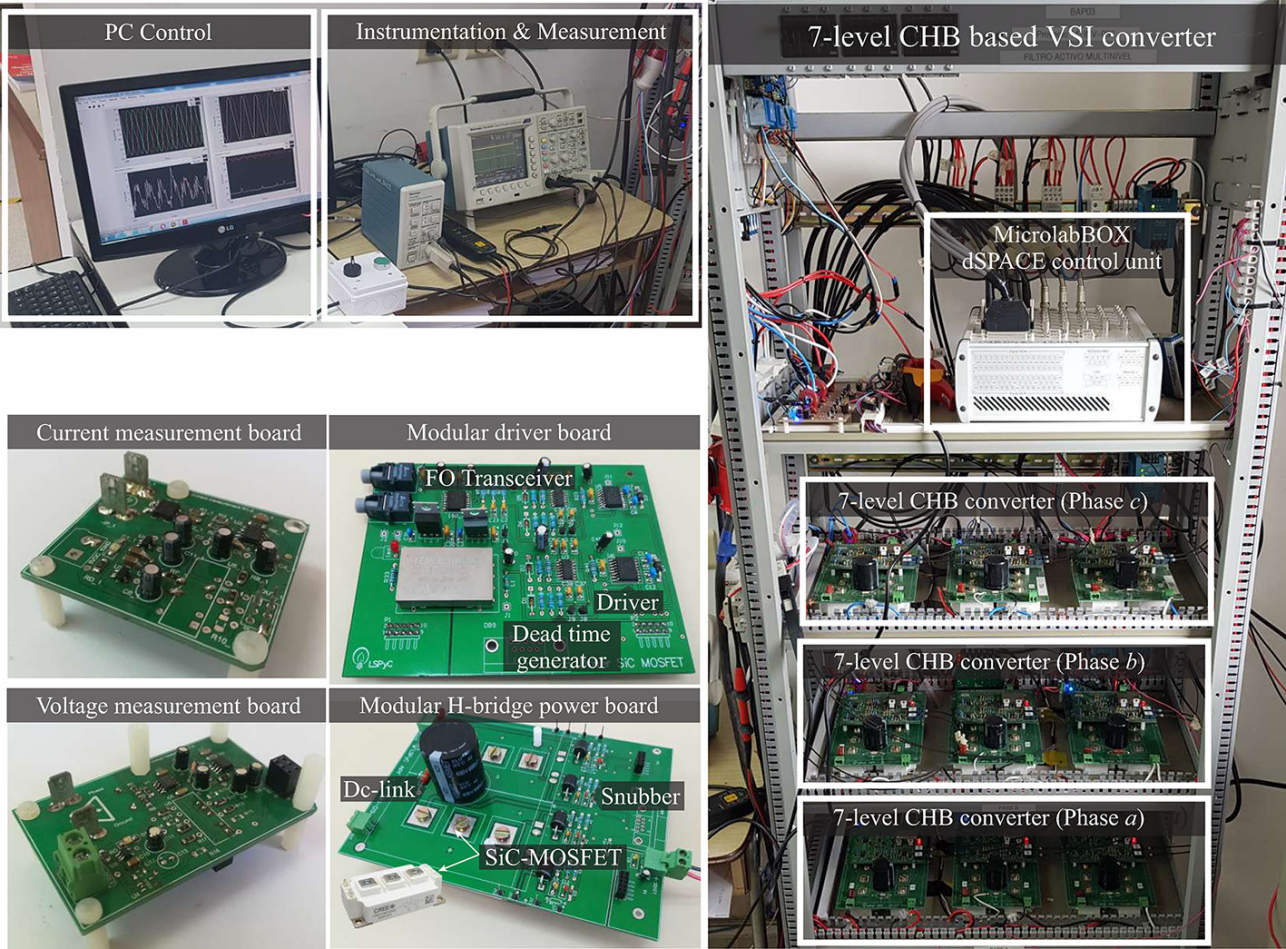
Mounting the 7-level CHB converter.

Fig. 19 shows the final assembly of the 7-level CHB converter, in which the main elements are observed: the H-bridge cells (power board and controller), voltage and current sensors, the controller device and finally, the PC where the measurements are displayed in real-time. Each of the boards mentioned has a modular and scalable design, in such a way as to facilitate changes in the converter configuration according to the end user’s requirements. In addition, this modular structure facilitates the replacement of damaged parts.

Regarding the designs of printed circuits, it can be mentioned that all the integrated circuits (IC) used to assemble them are SOIC-type surface mounts. For cases in which there are no integrated libraries of their own, they were designed to make up for that lack. The schematic diagram and PCB layout were developed using the Altium Designer application. The designed boards are routed to two layers to reduce the dimension of the resulting board, different ground planes were used on both sides for digital and analogue grounds, in addition to implementing galvanic isolation towards the controller side, thus reducing interference. generated during the switching process of the H-bridge cells. The trigger PWM signals are sent through an individual optic fibre link, from the controller GPIO modules to an optic fibre transmitter board, these signals are then processed on the controller boards of each H-bridge.

Before the tests and calibration process of each board, all the tracks of each of the boards were subjected to quality tests, verifying the continuity and insulation of each section. For the assembly process of each board, a standard procedure was followed, where the first to be assembled was the surface solder elements, followed by the through-encapsulation components and finally the connectors. Component names are clearly labelled on the PCB silkscreen and correspond to the names on the material lists.

Finally, for the assembly of the power converter hardware, a reinforced metal structure with a capacity for six levels is selected, where each level is a metal tray that is used as a base to house instrumentation equipment, sensors and the H-bridge cells, with cascade arranged.

## 6 Operation instructions

**Fig. 20:**
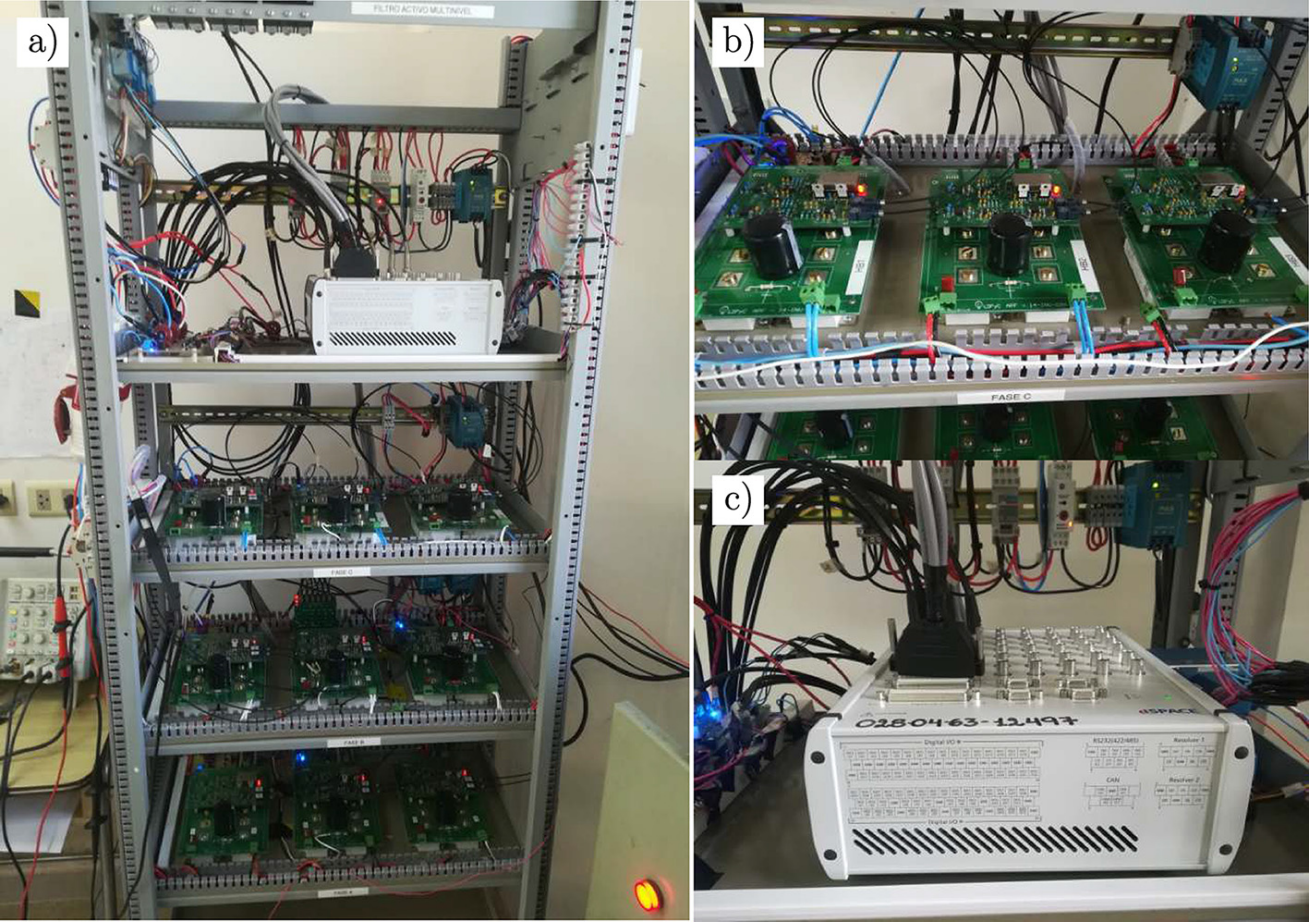
Integration of the different designed modules. a) Front view of the multilevel converter, b) H-bridge cells and c) dSPACE controller.

Fig. 20 shows the different modules, which make up the H-bridge cells, the instrumentation, protection and control equipment, as well as the phase voltage and current adaptation plates.

**Fig. 21:**
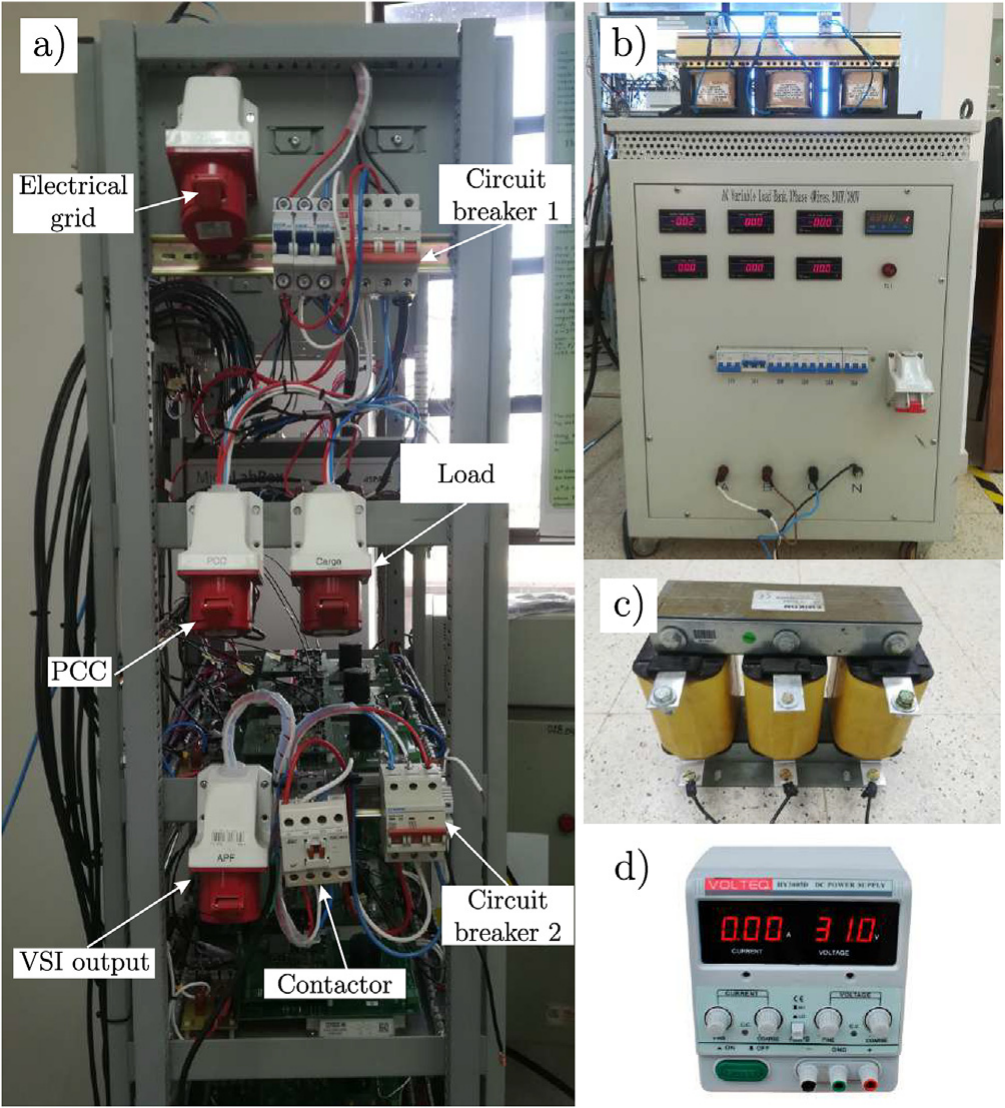
a) Side view of the multilevel converter, b) Load RL, c) Output R-L filter and d) DC-link capacitors.

On the other hand, in the photograph of Fig. 21 (a), shows the three-phase sockets corresponding to the connection of the multilevel converter, which consists of a 4-pole three-phase connector with a capacity of 16 A together with its 16 A thermomagnetic protection key (Circuit Breaker 1). Likewise, the connectors correspond to the output of the multilevel converter, which has its own protection consisting of a 16 A three-phase thermomagnetic key (Circuit Breaker 2). Fig. 21 (b) shows the three-phase test load used for the experimental tests, Fig. 21 (c) presents the output filter for the converter, and finally Fig. 21 (b) shows one of the DC sources used for the DC-link.

The recommended procedure for operating the converter is as follows:

• Connect the load for use in the three-phase output socket (VSI output).
• Connect the mains power to the corresponding three-phase socket (Electrical grid)
• Activate Circuit Breaker 1 to power the converter
• Turn on the controller (MicrolabBox) and the data monitoring PC.
• Activate Circuit Breaker 2 for protection at the output of the power converter.
• Activate the output contactor to supply the three-phase load.

Among the precautions to be taken into account during the start-up process, we have the verification of the correct connection to the electrical network so that the circuits and control and measurement are all in an operational state, for this, each designed board has an indicator light for quick visual inspection.

## 7 Validation and characterization

### 7.1 Analysis of the H-bridge cell

**Fig. 22:**
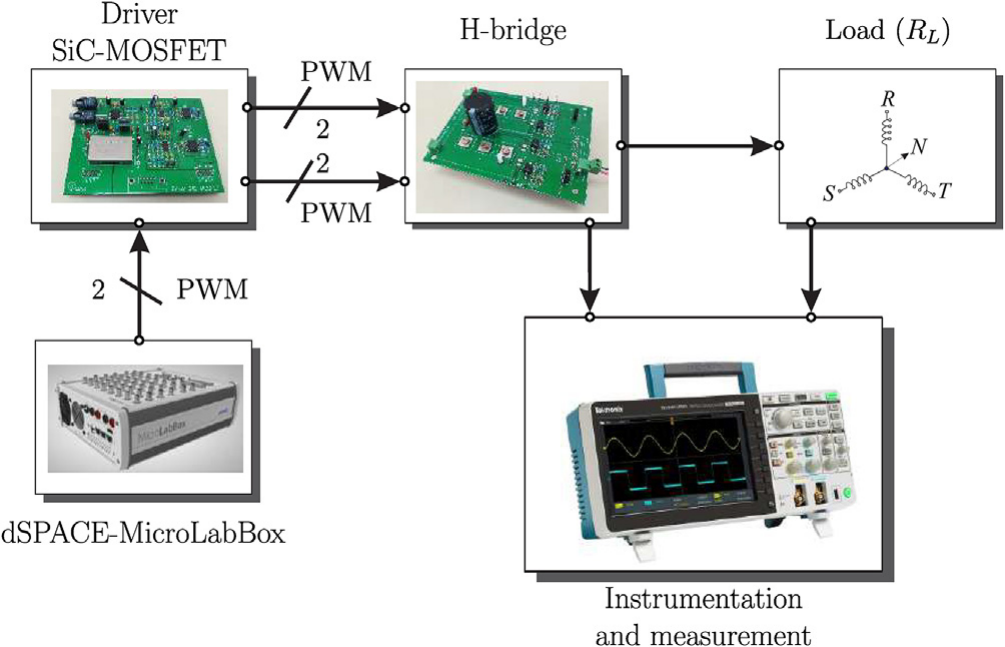
Test platform schematic for controller and H-bridge.

The test platform for the H-bridge converter is shown in Fig. 22. The scheme consists of two independent modules, which are the controller board or driver for the SiC-MOSFETs and the power board. In addition, a MicroLabBox-dSPACE controller is used for the implementation of the control algorithms and generation of PWM trigger signals. The output signals are measured on the load and captured through the use of digital oscilloscopes. In this context, the specifications of the experiment, designed to verify compliance with the proposed design objectives, are shown in Table 4.

**Table 4:** Technical parameters for experiments.

Parameter	Value	Unit
DC-link	30	V
Load	56	Ω
fs	20	kHz

**Fig. 23:**
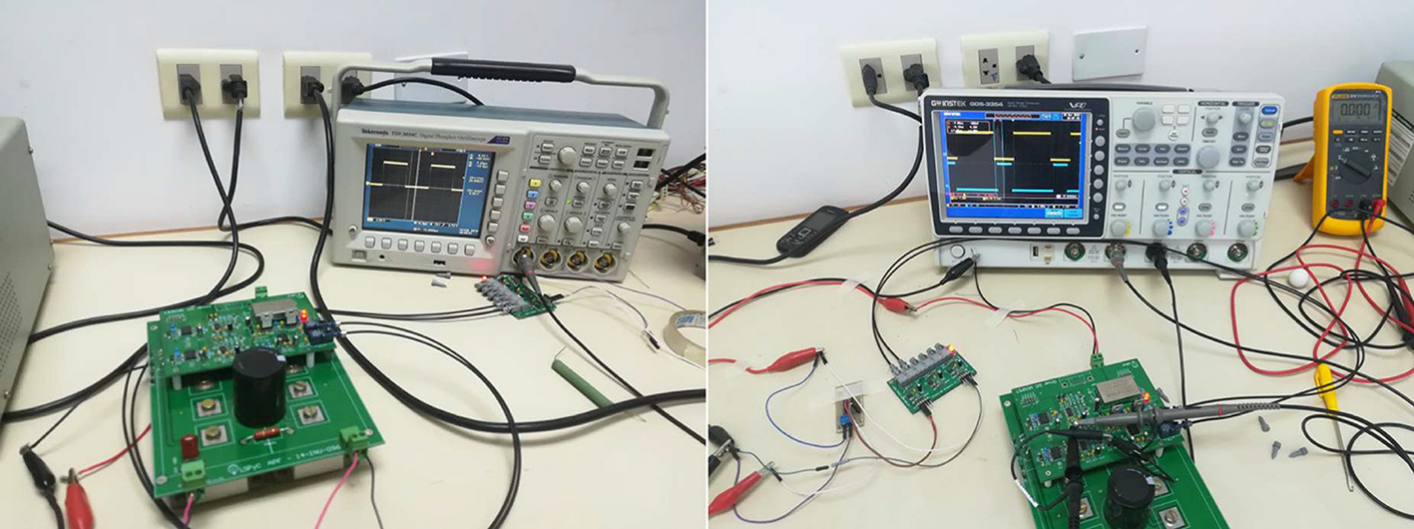
Test platform implemented in the laboratory.

Fig. 23 shows the experimental test platform to obtain the preliminary results of the H-bridge cell, used to characterise the design proposal to be implemented in the multilevel VSI.

**Fig. 24:**
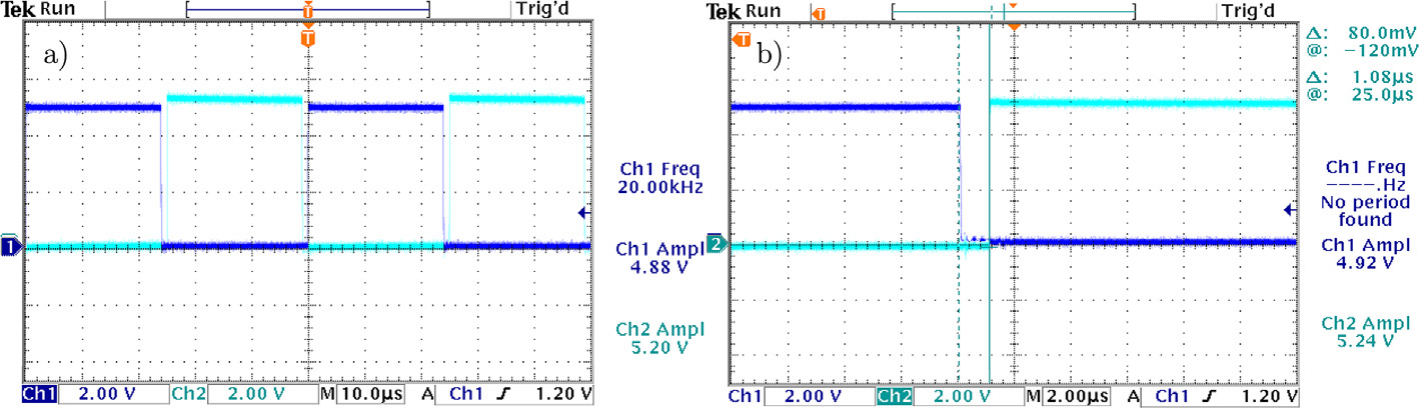
Complementary trigger signals for the H-bridge. a) Complementary trigger signals for the H-bridge. b) Dead time between complementary signals.

The trigger signals corresponding to each SIC-MOSFET that make up the VSI are represented in Fig. 24 (a). Those trigger signals are obtained at the outputs of the complementary signal generation circuits and dead time present on the firing control board. According to the results, the generated signals present good electrical characteristics considering the low level of noise observed and the delays due to the settling times of the signals. The Fig. 24 (b) shows the result of the generation of dead time by hardware. It has a value of 1 μs for a frequency of 20 kHz.

**Fig. 25:**
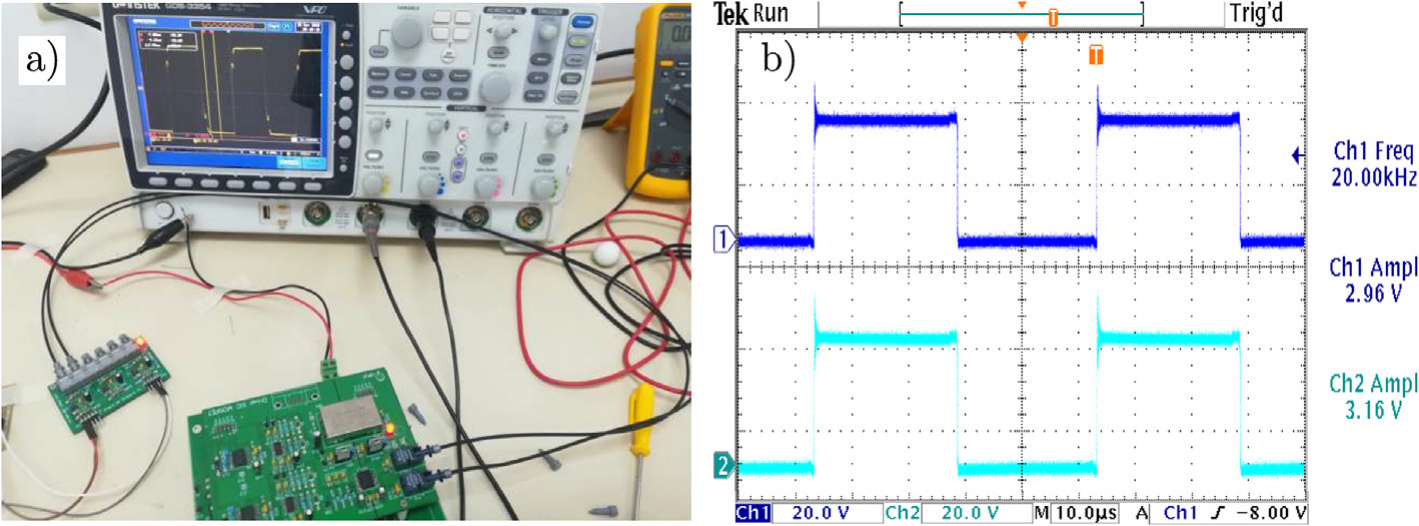
a) Test platform for the H-bridge. b) H-bridge output signal.

It is important to mention that the dead time value can be adjusted according to the switching frequency to be used. This time is essential to avoid the momentary short-circuit existing between the activation and deactivation of the switches of the same leg of the VSI, and this in turn would generate power losses and a decrease in the performance of the system. The power board consists of the signal conditioning circuits for each SIC-MOSFET, consisting of passive filters for damping overshoots that could occur in the signals coming from the controller integrated circuits for SIC-MOSFETs. In order to facilitate the analysis of the scheme, relevant measurement points are added. The DC link to be used for the design has a maximum value of 480 V. Fig. 25(a) shows the PCB layout for the power board.

The output signals for the H-bridge cell are shown in Fig. 25(b). These output signals are essential to guarantee proper operation of the switching system and ensure effective current and voltage control at the load. The obtained results adequately comply with the proposed design, according to captured signals by the oscilloscope.

### 7.2 Voltage Sensor Validation

**Fig. 26:**
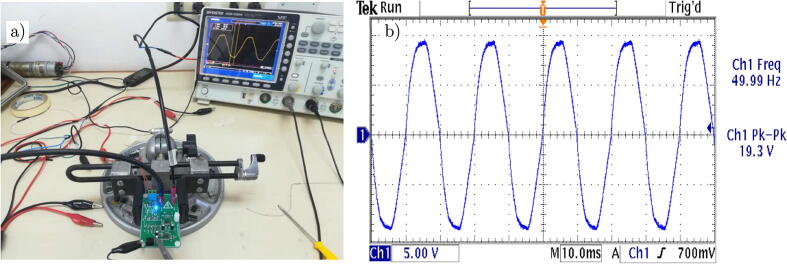
a) Test platform for the voltage sensor. b) Voltage output signal of the designed conditioning circuit.

Fig. 26 (a) shows the test platform used in the laboratory for the experimental validation of the voltage sensor. It allows measuring AC voltages up to 380 Vrms. Fig. 26 (b) presents the captured waveform, by the oscilloscope, of the voltage signals.

### 7.3 Current Sensor Validation

**Fig. 27:**
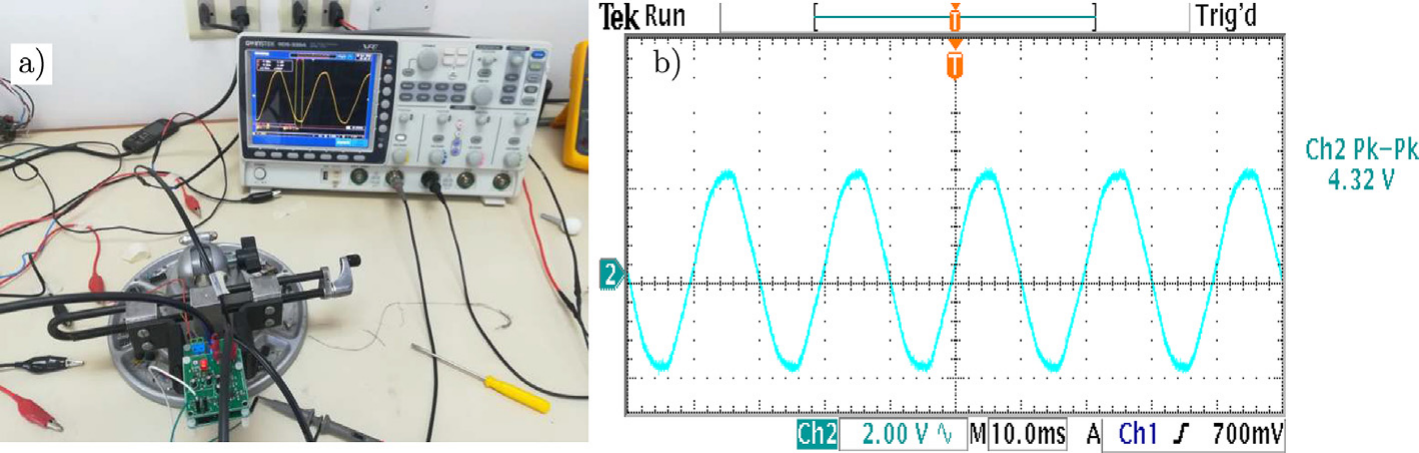
a) Test platform for the current sensor. b) Current output signal of the designed conditioning circuit.

Fig. 27(a) shows the test platform used in the laboratory for the experimental validation of the current sensor. Fig. 27(b) presents the evolution of the measured current when using a resistive load.

**Fig. 28:**
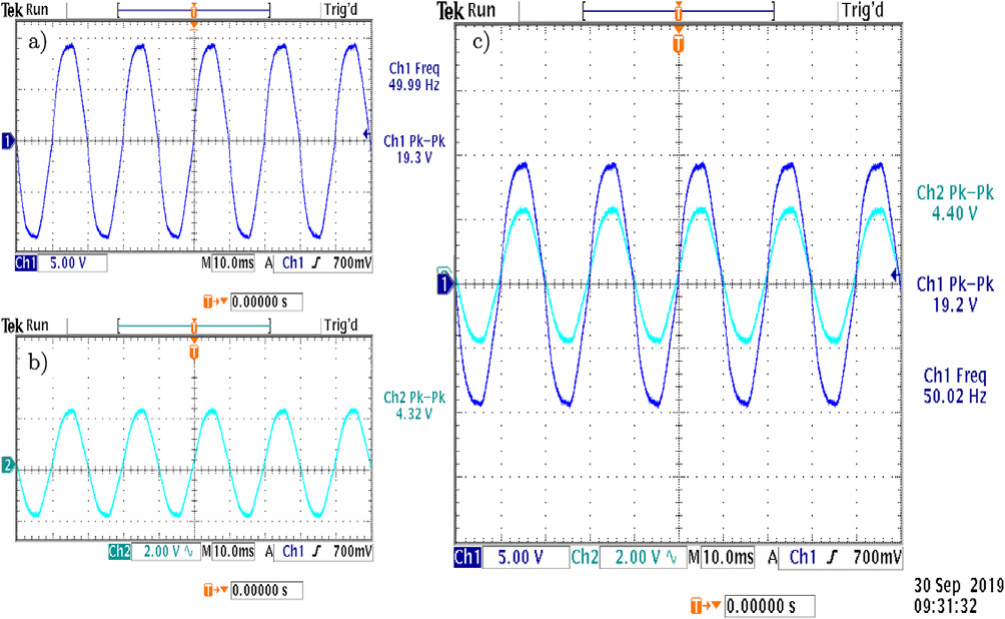
Output signals of the measurement circuits. a) Measured voltage, b) Measured current, c) Signal superposition and phase shift.

Finally, Fig. 28 shows the superposition of both measurements, observing a negligible phase shift between the voltage and current measurements on the load, which is a desirable characteristic at the moment of the implementation of control algorithms.

### 7.4 Experimental results of the multilevel VSI

**Fig. 29:**
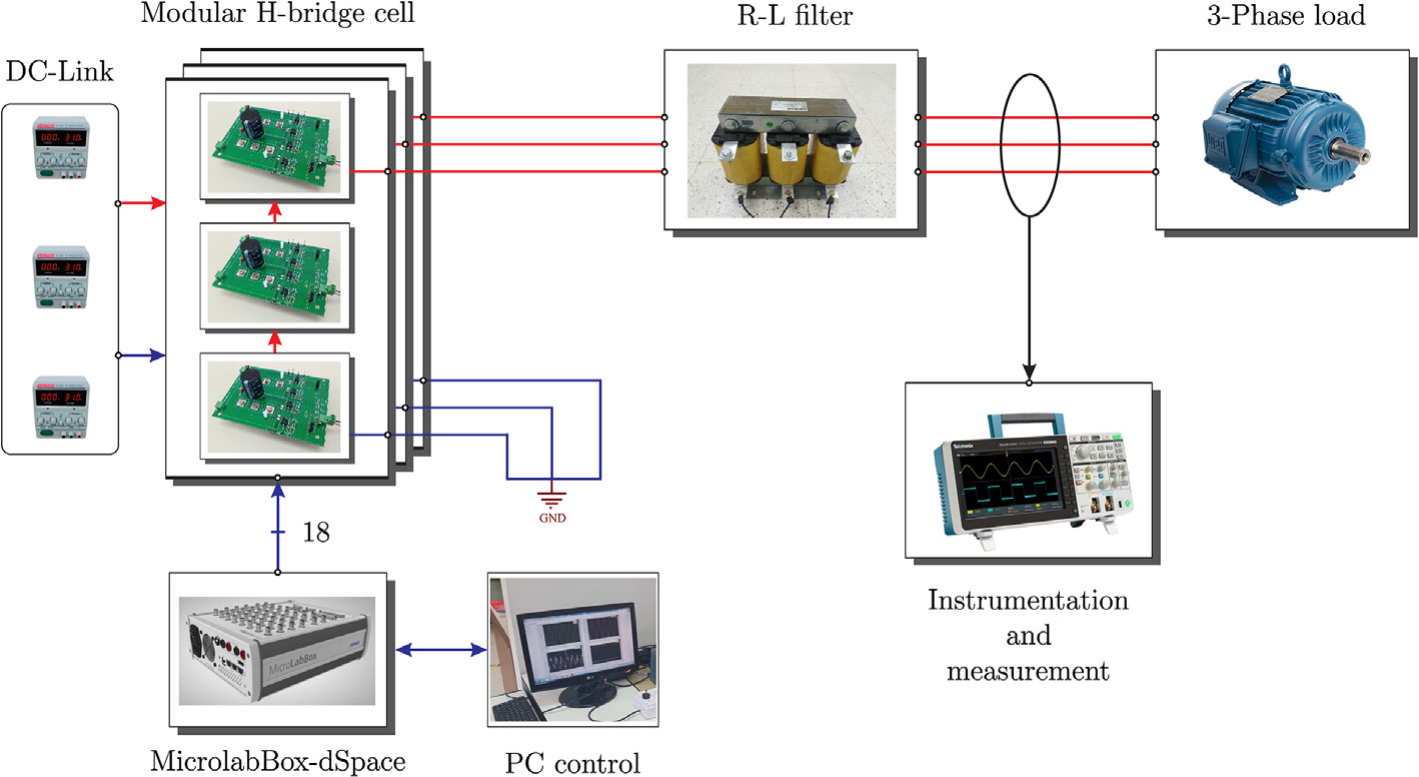
Scheme used to evaluate the multilevel VSI experimental platform.

The experimental platform of the multilevel VSI is made up of H-bridge cells interconnected in cascade, where each H-bridge cell consists of an independent DC-link that is applied through continuous sources and provides the necessary energy to the switching devices for the generation of output voltages that are injected into the load at the common connection point (PCC). In this mode of operation, series-connected SiC-MOSFET switching devices and an R-L inductive filter are integrated into the VSI’s output. This filter reduces the effect of the harmonic components resulting from the switching of the power devices that make up the VSI. Fig. 29 denotes the configuration of the experimental platform for use in multilevel VSI mode.

**Fig. 30:**
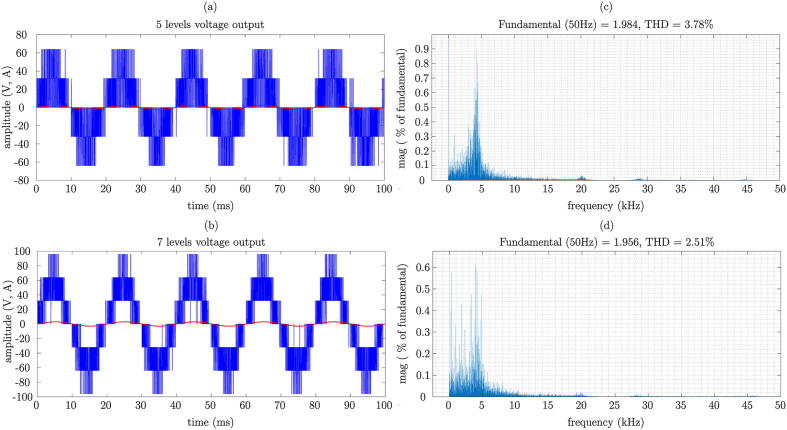
THD analysis based on simulation results. a) 5 levels, b) 7 levels.

For this paper, a preliminary analysis was conducted using simulation tools for the multilevel converter with 5-level and 7-level voltage schemes prior to experimental measurements. The obtained results from the simulation are shown in Fig. 30. Fig. 30(a) and Fig. 30(b) display the output voltage and current waveforms of the converter for the cases of 5 levels and 7 levels, respectively. Fig. 30(c) and Fig. 30(d) show the levels of total harmonic distortion (THD).

For the 5-level converter, a THD of 3.78% is obtained, while the 7-level converter exhibits a THD of 2.51%. It can be observed that the 7-level converter has a lower harmonic content, as indicated by previous studies.

To verify the proper functioning of the designed power converter, experimental results are obtained from the multilevel VSI test platform, in which a predictive current control is implemented. To achieve the proposed objective, it is initially considered that the VSI regulates the current injected into a pure resistive three-phase load, also assuming the following parameters; DC-link voltage, Vdc  = 30 V, switching frequency $f_{s}$ = 33 kHz and load $R_{L}$ = 16Ω.

**Fig. 31:**
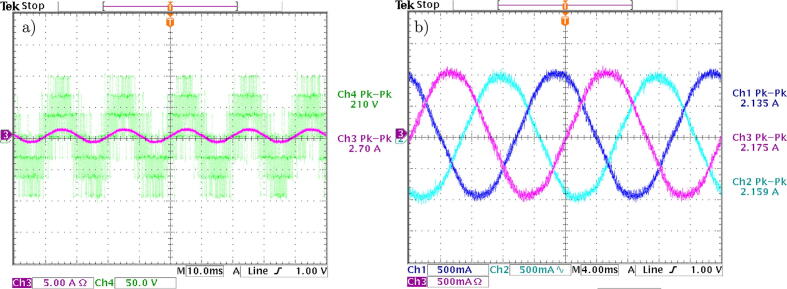
Experimental results of the multilevel VSI with MBPC. a) Multilevel VSI output signals, b) Multilevel VSI output currents.

The output signal of the power converter $v_{c}^{\phi }$, as well as the generated current for the phase $i_{c}^{\phi }$ from the references and as a result of implementing a classic predictive control without modulation, is seen in the oscilloscope screenshot, shown in Fig. 31(a). On the other hand, the obtained three-phase currents by the converter in multilevel VSI mode are observed in Fig. 31(b). It can be seen in these graphs that the applied currents to the three-phase load are generated in the correct sequence, yielding a low harmonic distortion content, which is to be expected thanks to the ability of the multilevel converter to generate sinusoidal signals.

One of the most important sources of harmonic generation in electrical installations is power converters. To properly understand the adverse effects on the electrical system, it is important to determine the level of harmonics introduced by the proposed VSI design [19]. For this, a study of the total harmonic distortion (THD) level introduced by the converter is carried out through the obtained measurements from the current and voltage signals. This THD level result was obtained using the fast Fourier transform, an oscilloscope function. Mathematically speaking, THD is defined as:

(6) $$\mathrm{THD}(i_{s})=\sqrt[{}]{\frac{1}{i_{s1}^{2}}\overset{N}{\underset{j=2}{\sum }}{\left(i_{\mathrm{sj}}\right)}^{2}}$$

where $i_{s1}$ is the fundamental component of the measured currents and $i_{\mathrm{sj}}$ are the harmonic currents.

**Fig. 32:**
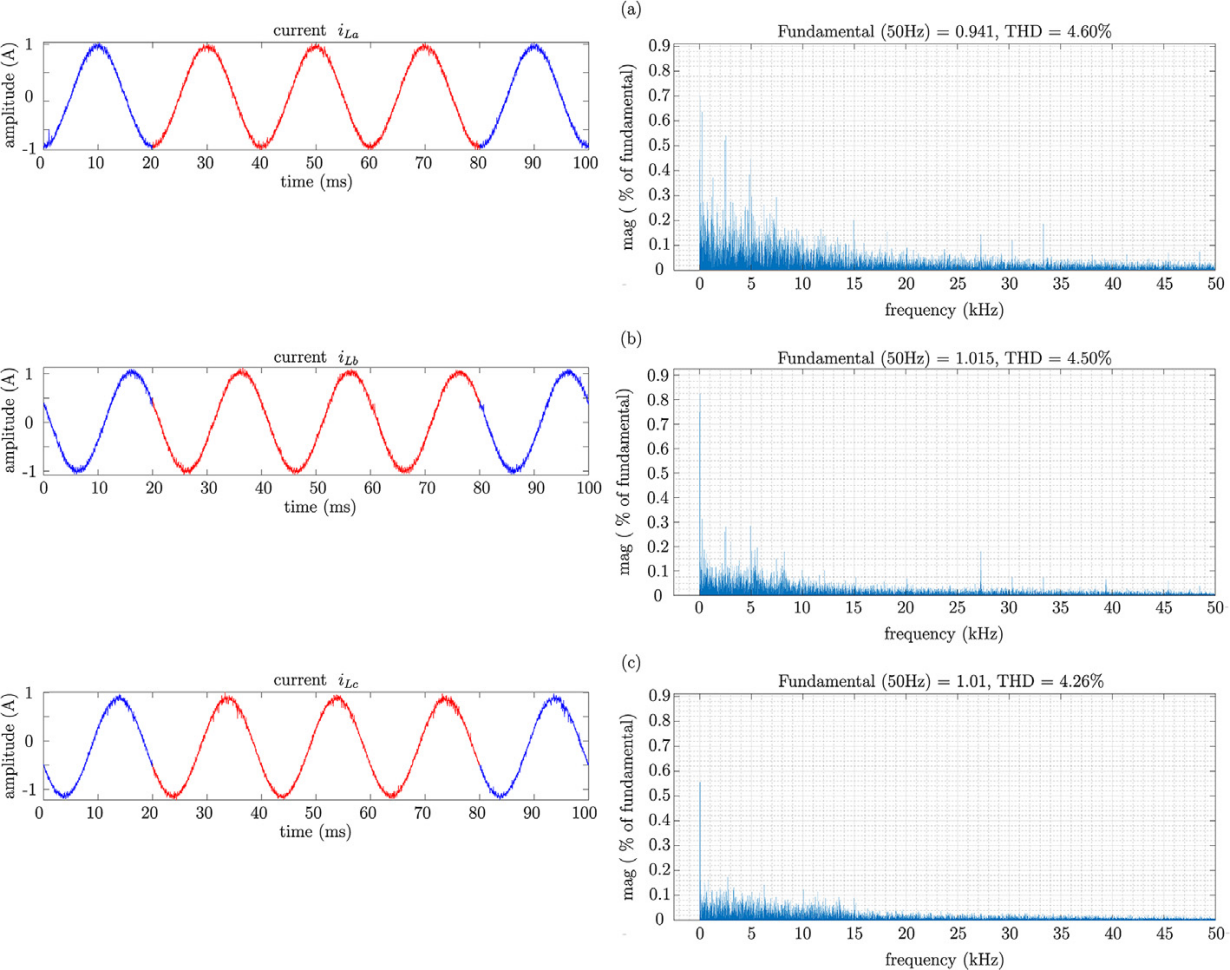
THD analysis based on experimental results. a) Phase a, b) Phase b, c) Phase c.

For the experimental validation of the design, the analysis of Total Harmonic Distortion (THD) of the system has been conducted for the case of the 7-level converter. The THD has been measured in each phase of the multilevel converter. Fig. 32 shows the results from the calculations of the level of THD of the output current of the multilevel VSI, applied to the load. Fig. 32 presents similar levels of THD results for each phase, with values of 4.60% for phase a, 4.50% for phase b, and 4.26% for phase c.

**Fig. 33:**
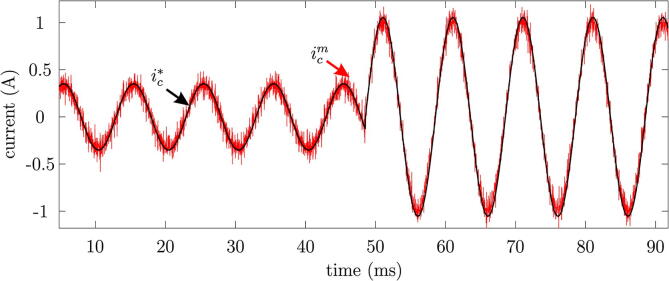
Measured output current for a change in reference amplitude.

Subsequently, a study of the dynamic behaviour of the multilevel converter is carried out, adjusting parameters representative of the desired output signal, such as the frequency and amplitude of the output current. Fig. 33 shows the obtained results in the output signals of the multilevel VSI with reference changes in the amplitude in the implemented control algorithms.

It is observed that the converter presents a good dynamic response to the change of reference amplitude, managing to modify such amplitude in a considerably short time. The calculated mean square error (MSE) between the reference signal and the measured value is 0.081 A.

**Fig. 34:**
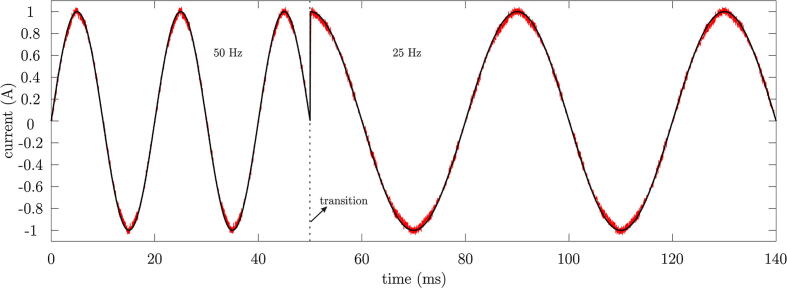
Measured output current for a change in reference frequency.

On the other hand, Fig. 34 shows the obtained results in the output signals of the multilevel VSI before changes in the frequency of the generated reference. A good dynamic response of the converter to rapid changes in the reference frequency is also observed. Also, for the dynamic analysis of the reference frequency change, the MSE analysis between the reference and the measurement was performed, resulting in a value of 0.08 A.

Finally, the efficiency of the designed H-bridge cells was calculated by determining the power losses due to conduction and switching for an individual H-bridge cell based on SiC-MOSFET devices. The following equations are used to determine conduction losses (7) and switching losses (8).

(7) $$P_{\mathrm{CM}}=V_{\mathrm{DS}}\;I_{\mathrm{Drms}}=R_{\mathrm{DS}(\mathrm{on})}\;I_{\mathrm{Drms}}^{2}$$

(8) $$P_{\mathrm{sw}}=\frac{V_{\mathrm{DD}}\;I_{D}}{2}(t_{\mathrm{on}}+t_{\mathrm{off}})f_{s}$$

being $I_{\mathrm{Drms}}$ the drain current root-mean squared (rms), $f_{s}$ the operating frequency, $V_{\mathrm{DD}}$ the DC-link voltage, $R_{\mathrm{DS}(\mathrm{on})}$ the drain source resistance, $t_{\mathrm{on}}$ and $t_{\mathrm{off}}$ the state transition time intervals, turn-on and turn-off, respectively.

**Fig. 35:**
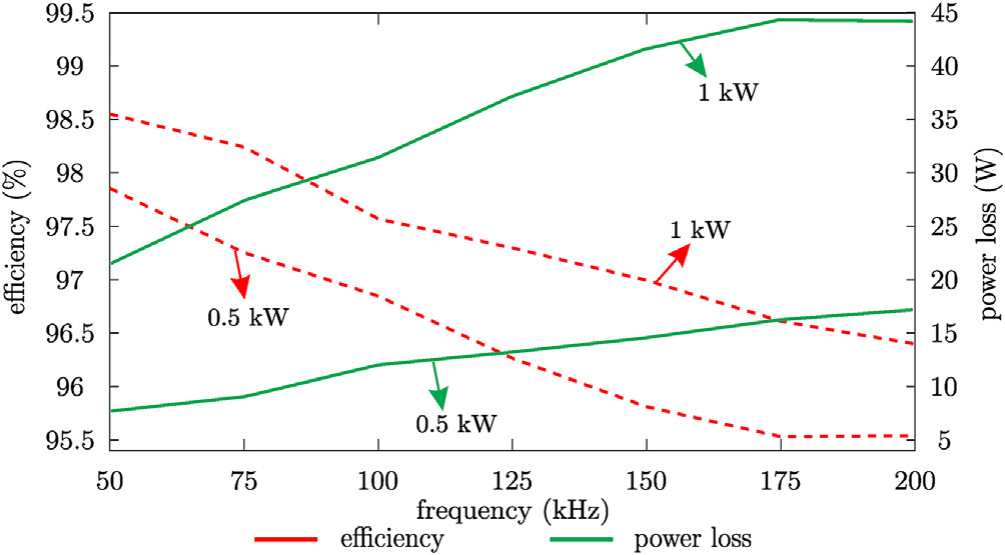
Efficiency (red lines) and power losses (green lines) regarding the switching frequency.

Laboratory tests were performed for an individual H-bridge cell, two power values, 0.5 kW and 1 kW, were considered for the test. In turn, the switching frequency range of 50 kHz to 200 kHz was analyzed. As observed in Fig. 35, the power losses increase with frequency, and with it the efficiency decreases, maintaining an efficiency higher than 95% in all cases.

## 8 Conclusion

This article has addressed in detail the design and then the experimental validation of different electronic circuits that make up the multilevel power electronic converter based on H-bridge cells with a modular structure. For the design of each circuit, the recommendations are present in the design guidelines, aligned with the datasheets of the manufacturers of the different electronics employed.

Regarding the experimental results, tests were carried out for all the proposed designs to corroborate the correct functioning of the circuits and ensure that they meet the design objectives. Calibration tests of the voltage and current sensors were carried out, as well as the experimental verification of the correct operation of the firing control circuits of the SiC-MOSFETs of each H-bridge cell.

A seven-level voltage source inverter has been designed to synthesise a three-phase alternating current output of the indicated value by its reference with a relatively low THD percentage of 4.45% on average, resulting in a value lower than the maximum recommended by the quality standards. In addition, tests were carried out to determine the correct dynamic operation of the converter through changes in the amplitude of the reference, as well as in the desired output frequency. These analyses gave a satisfactory result. As for the energy efficiency of the designed H-bridge cell, the efficiency is greater than 95% according to the experimental tests carried out for operating frequencies between 50 kHz and 200 kHz, noting that the efficiency decreases as the operating frequency increases due to losses caused by switching.

## CRediT authorship contribution statement

**Julio Pacher:** Conceptualization, Methodology, Software, Visualization, Writing - original draft. **Jorge Rodas:** Funding acquisition, Project administration, Data curation, Writing - original draft. **Alfredo Renault:** Conceptualization, Methodology, Supervision, Visualization. **Magno Ayala:** Writing - review & editing. **Leonardo Comparatore:** Writing - review & editing.

## Declaration of Competing Interest

The authors declare that they have no known competing financial interests or personal relationships that could have appeared to influence the work reported in this paper.
